# Anti- Melanoma Differentiation-Associated Gene 5 Antibody Positive Dermatomyositis: Recent Progress in Pathophysiology and Treatment

**DOI:** 10.1007/s11926-025-01188-7

**Published:** 2025-05-05

**Authors:** Tsuneyasu Yoshida, Ran Nakashima

**Affiliations:** 1 Department of Rheumatology and Clinical Immunology, Graduate School of Medicine, Kyoto University, Kyoto, Japan; 2 Division of Clinical Immunology and Cancer Immunotherapy, Center for Cancer Immunotherapy and Immunobiology, Graduate School of Medicine, Kyoto University, 54 Shogoin-Kawahara-cho, Sakyo-ku, Kyoto, 606-8507 Japan

**Keywords:** MDA5, Anti- Melanoma differentiation-associated gene 5 antibody positive dermatomyositis, Pathogenesis, Mechanism

## Abstract

**Purpose of Review:**

Anti-melanoma differentiation-associated gene 5 (MDA5) antibody-positive dermatomyositis (MDA5-DM) is a rare systemic autoimmune disease characterized by a clinically amyopathic presentation and a high-risk association with rapidly progressive interstitial lung disease. Although frequently fatal, the underlying mechanisms remain incompletely understood. This review provides a comprehensive summary of recent advances in research on MDA5-DM, aiming to deepen our understanding of its pathogenic mechanisms and to accelerate future basic research that will contribute to the development of novel therapeutic strategies.

**Recent Findings:**

Recent advancements have shed light on various aspects of this disease, including genetic and environmental factors contributing to disease susceptibility and the immunopathological processes and cytokine networks. Furthermore, significant progress has been made in understanding the pathogenicity, epitope recognition, and production mechanisms of anti-MDA5 antibodies, which have long been subjects of debate. On the therapeutic front, in addition to the conventional triple-combination regimen, emerging efficacy of JAK inhibitors and rituximab has been recognized. The development of biologics targeting lymphocytes offers additional hope for advancing therapeutic options.

**Summary:**

Advancing our understanding of the latest pathophysiological mechanisms of MDA5-DM is expected to pave the way for the development of safer and more effective therapeutic strategies.

**Supplementary Information:**

The online version contains supplementary material available at 10.1007/s11926-025-01188-7.

## Introduction

Anti-melanoma differentiation-associated gene 5 (MDA5) antibody-positive dermatomyositis (MDA5-DM) is a systemic autoimmune disease characterized by inflammation of the skin, lungs, and vasculature. While it accounts for less than 2% of dermatomyositis cases in Caucasian populations, it comprises over 20% in Asian populations—more than ten times higher than in Caucasian [1–3]. Moreover, the overall prevalence of MDA5-DM is notably higher in Asians compared to Caucasians (7–16%) [4].

A hallmark feature of MDA5-DM is its prevalence of the clinically amyopathic dermatomyositis (CADM) phenotype, characterized by minimal or absent muscle involvement. Additionally, the disease is frequently complicated by rapidly progressive interstitial lung disease (RP-ILD), which is often refractory to treatment and associated with a 6-month mortality rate of 28–66%, making it one of the most life-threatening autoimmune diseases [5–7]. Notably, the incidence of this fatal RP-ILD shows marked ethnic variation: while 50–100% of Asian patients with MDA5-DM develop RP-ILD, the frequency is considerably lower in Caucasians, ranging from 20 to 57% [7–12].

Although there is an urgent need for novel therapeutic strategies to treat RP-ILD associated with MDA5-DM, our understanding of its pathogenesis remains incomplete. However, recent years have witnessed a growing body of research—particularly in basic science—beginning to illuminate the complex mechanisms underlying this disease.

This review aims to provide a comprehensive overview of the latest advances in MDA5-DM research, with the goal of deepening our understanding of its pathophysiology and fostering the development of novel therapeutic approaches.

## Factors Contributing to MDA5-DM

Recent advances in research have suggested that, similar to other rheumatic diseases, the development of MDA5-DM results from a complex interplay of genetic and environmental factors. On the genetic front, specific gene variants associated with susceptibility to MDA5-DM have been identified, with notable differences in the prevalence and expression of these variants across ethnic groups. As for environmental triggers, certain viral infections have emerged as potential contributors to disease onset and are increasingly being recognized as key inciting factors.

### Genetic Factors

In terms of genetic factors contributing to MDA5-DM, earlier studies from Japan have demonstrated an association between disease susceptibility and specific human leukocyte antigen (HLA) alleles—namely HLA-DRB1*0101 and *0405 [13]. HLA-DR, a component of the major histocompatibility complex (MHC) class II, plays a crucial role in presenting antigenic peptides from antigen-presenting cells to T cells. These HLA molecules are critical in autoimmune diseases because they facilitate the presentation of self-antigens to the immune system, thereby promoting autoantibody production.

For instance, in rheumatoid arthritis, certain HLA haplotypes and alleles are known to have a strong binding affinity to citrullinated peptides, which facilitates their presentation to T cells and is associated with the production of anti-cyclic citrullinated peptide antibodies. Similarly, in MDA5-DM, HLA-DRB1*0101 and *0405 alleles may predispose individuals to the disease by presenting MDA5-derived peptides to T cells, thereby contributing to the development of anti-MDA5 antibodies.

Interestingly, studies in Caucasian populations have not identified HLA alleles significantly associated with MDA5-DM [14]. As discussed later, there are known differences in the MDA5 epitope regions recognized by anti-MDA5 antibodies across ethnic groups. These discrepancies in HLA haplotypes may influence both the prevalence of anti-MDA5 positivity and the repertoire of targeted epitopes, potentially contributing to the observed racial differences in MDA5-DM susceptibility and clinical phenotype.

Among HLA, genome-wide association studies have identified a truncated variant (rs7919656) of the *WDFY4* gene that is associated with both CADM and RP-ILD [15, 16]. While conventional dendritic cells typically present antigens to CD4⁺ T cells, WDFY4 appears to facilitate cross-presentation, enabling antigen presentation to CD8⁺ T cells as well [17] potentially contributing to their activation. Moreover, WDFY4 has been reported to amplify MDA5-mediated NF-κB signaling [15] and regulate CD8⁺ T cell apoptosis through a p53 signaling [18]. These findings suggest that *WDFY4* mutations or functional alterations may play a role in promoting autoimmune responses.

Although the WDFY4 variant has not been detected in European populations, it has been reported in East Asian patients, including Japanese and Chinese individuals with MDA5-DM, and is reported to be associated with RP-ILD [15, 16]. Together with HLA differences, these population-specific genetic variants may help explain the distinct prevalence and severity of MDA5-DM between Caucasian and Asian populations [19].

## Environmental Factors

A tendency for MDA5-DM to occur more frequently among individuals residing in suburban areas or near rivers has long been recognized [6]. More recently, a seasonal pattern has also been observed, with disease onset peaking from winter to spring across different ethnic populations [6, 20–23]. These geographic and seasonal trends suggest that environmental factors may play a role in the pathogenesis of MDA5-DM. Given that MDA5 functions as an intracellular viral sensor capable of recognizing a wide range of viruses, viral infections have emerged as a plausible environmental trigger for disease onset.

Upon recognizing viral RNA, MDA5 initiates the production of type I interferons (IFNs), which exert antiviral effects. Interestingly, type I IFNs also upregulate MDA5 expression itself [24–26], suggesting the existence of a positive feedback loop in which viral infection activates MDA5, leading to increased type I IFN production, which in turn further enhances MDA5 expression. In MDA5-DM, this feedback loop may become localized to inflamed tissues such as the lungs and skin, thereby contributing to the characteristic organ damage observed in the disease.

An integrated miRNA–mRNA association analysis using circulating monocytes from MDA5-DM patients has revealed that upstream regulators of the type I IFN response—specifically TLR3, TLR7, and TLR9, which recognize viral genetic material—are transcriptionally regulated by PU.1. This regulation promotes both type I IFN–mediated inflammation and the upregulation and secretion of CCL2, ultimately driving antiviral immune responses [27]. These findings suggest that infections with single- or double-stranded RNA viruses, which serve as ligands for TLR3/7/9, may activate these upstream regulators and contribute to the pathogenesis of MDA5-DM.

Although MDA5 is primarily known to recognize picornaviruses, direct evidence linking specific viral infections to MDA5-DM has been limited. However, recent gene set enrichment analyses have shown enhanced expression of genes related to the herpes simplex virus type 1 (HSV-1) infection pathway in patients with dermatomyositis-associated interstitial lung disease. Furthermore, MDA5-DM patients exhibit increased expression of genes that inhibit HSV-1 protein synthesis, suggesting heightened immune responses against HSV-1 in this population [28]. In addition, another study using sera from MDA5-DM patients reported elevated antibody responses against enterovirus B–derived peptides [29].

**Fig. 1:**
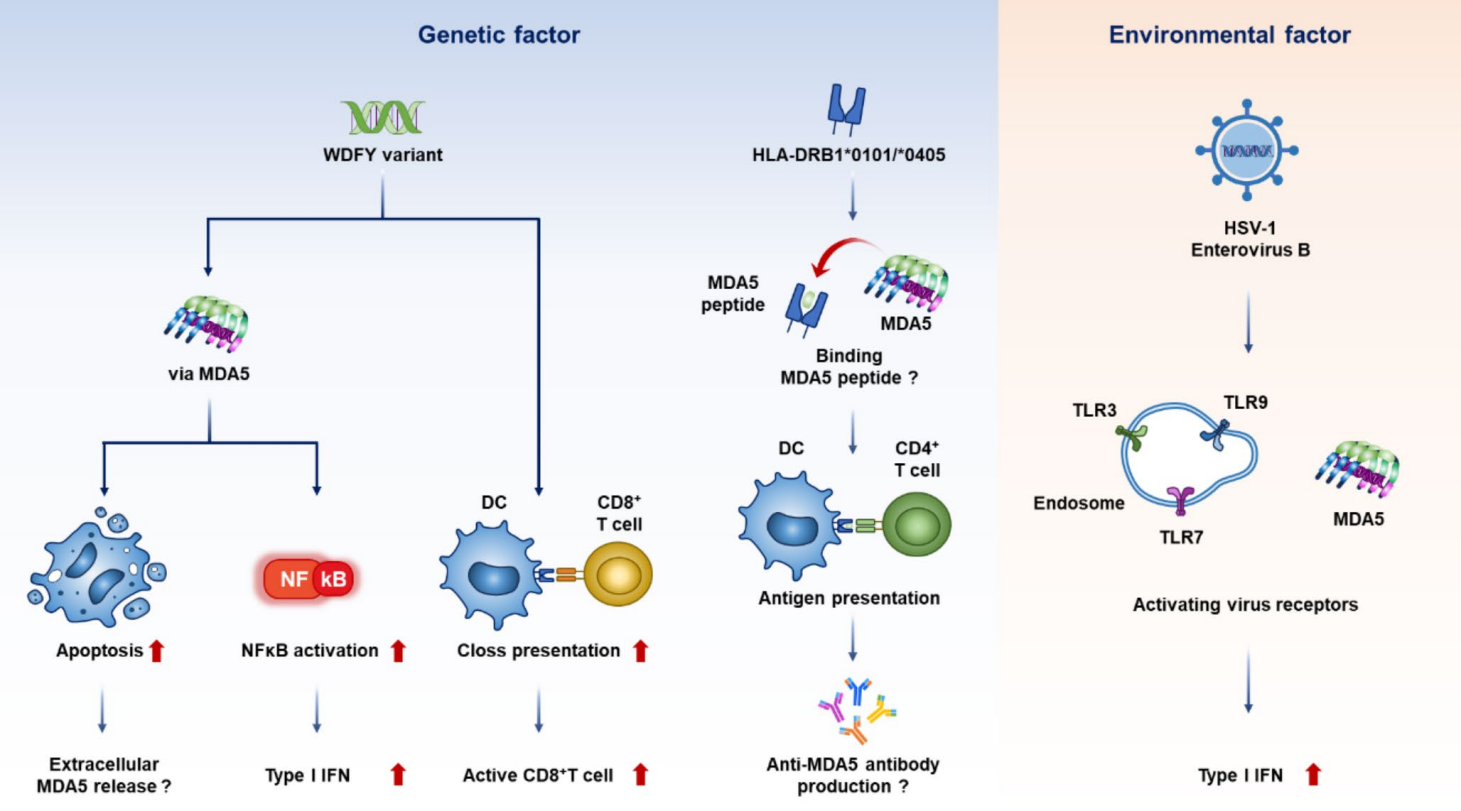
Genetic and environmental factors associated with MDA5-DM. DC: dendritic cell, IFN: interferon, MDA5: melanoma differentiation-associated gene 5, TLR: toll like receptor. This figure was originally created by the author using Microsoft PowerPoint (Office 2019)

Taken together, these findings suggest that certain viral infections—such as HSV-1 and enterovirus B—may act as environmental triggers in MDA5-DM by activating the type I IFN pathway through MDA5 and TLR signaling (Fig. 1).

## Immuno-pathophysiology of MDA5-DM

### Type I Interferonopathy and Macrophage Activation

Type I interferons (IFNs) play a central role in the pathogenesis of MDA5-DM. Not only is a type I interferonopathy signature consistently observed in patient sera [30, 31], but transcriptomic analyses have also revealed marked activation of type I IFN signaling in affected tissues—including the lungs, skin, and vasculature [32, 33]. This activation is closely linked to the frequent vascular damage seen in MDA5-DM [34] and is considered a key inflammatory trigger. Moreover, heightened type I IFN activity has been strongly associated with poor clinical outcomes in these patients [35].

Among the primary cellular sources of type I IFNs are macrophages and dendritic cells [36]. Notably, the activation of macrophages has been highlighted by proteomic analyses of patient sera, which have shown elevated levels of macrophage-derived or -associated proteins such as ferritin, chitotriosidase, soluble CD163, and Galectin-9. These molecules not only reflect macrophage activation but also serve as potential prognostic biomarkers [37–40]. In addition, inflammatory cytokines and chemokines associated with macrophage activation—such as CXCL10 (IP-10) and IL-34—are also elevated in MDA5-DM [41, 42]. Pathological analyses confirm the presence of activated macrophages within damaged tissues. Furthermore, single-cell RNA sequencing comparing immune cells from peripheral blood and bronchoalveolar lavage fluid (BALF) has demonstrated that macrophages within the lungs are the predominant producers of inflammatory cytokines, including type I IFNs [44].

### Type II and Type III Interferons

In addition to type I interferons (IFNs), elevated levels of type II IFN (IFN-γ) and type III IFN (IFN-λ) have also been reported in the sera of MDA5-DM patients [33, 43–45]. IFN-γ, primarily produced by T cells and NK cells, has been implicated in the development of RP-ILD in dermatomyositis (DM) [46]. Moreover, anti-MDA5 autoantibodies themselves have been shown to directly promote IFN-γ production in peripheral blood cells [47]. IFN-γ, in combination with IL-1β, induces the secretion of soluble CX3CL1 (fractalkine), a chemokine that attracts macrophages, from human lung fibroblasts [48, 49]. In the skin, IFN-γ stimulates keratinocytes to release IL-18, contributing to cutaneous pathology [50].

Among type III IFNs, IFN-λ3—known for its antiviral effects at epithelial barriers—has been identified as a predictor of disease activity and poor prognosis in patients with MDA5-DM, particularly those with skin and pulmonary involvement [45]. IFN-λ also promotes the differentiation of double negative 2 B cells via extracellular JAK/STAT1 signaling, a subset implicated in the pathogenesis of several autoimmune diseases, suggesting its involvement in MDA5-DM pathophysiology [51].

### T Cells

In patients with MDA5-DM, peripheral CD4⁺ and CD8⁺ T cells, as well as the CD4⁺/CD8⁺ ratio, are reduced in severe cases [52–54], and longitudinal decreases in T cells have been correlated with higher mortality [55]. Given the absence of lymphopoietic abnormalities in the bone marrow [52], this lymphopenia likely reflects either peripheral T cell death or recruitment into affected tissues.

In bronchoalveolar lavage fluid (BALF) from critically ill patients, activated T cells expressing IFN-stimulated genes such as ISG15⁺CD4⁺ and ISG15⁺CD8⁺ T cells have been observed to infiltrate lung tissues [33, 52]. T cell receptor (TCR) sequencing of peripheral blood and BALF samples has revealed shared clonotypes and clonal expansion in the lung, suggesting active recruitment of peripheral T cells to the lung [56].

Conversely, excessive activation of the RIG-I pathway in DM—including MDA5-DM—has been shown to induce T cell apoptosis and inhibit their proliferation in vitro [57]. Furthermore, single-cell transcriptomic analyses have revealed upregulation of genes involved in apoptosis, pyroptosis, and necrosis in peripheral T cells from severely ill patients [56], suggesting that T cell depletion may result from activation-induced cell death.

### B Cells

Serological and cellular analyses have shown clear evidence of B cell activation in MDA5-DM. Patients exhibit elevated serum levels of B cell-activating factor (BAFF) [58, 59], and CD19⁺ B cells are both increased and activated in peripheral blood [33, 60]. Subset analysis has shown a reduction in memory B cells and an increase in activated plasmablasts [61]. As in other autoimmune diseases, the coexistence of anti-Ro52 antibodies in MDA5-DM is associated with a higher frequency of RP-ILD and a worse prognosis [62–64]. In some patients, antibodies against SFPQ—a splicing factor implicated in innate immunity—have also been identified [65], suggesting a broader breakdown in B cell tolerance.

Ro52, an E3 ubiquitin ligase, targets IRF3 and IRF7 for ubiquitination, thereby downregulating type I IFN production [66]. The presence of anti-Ro52 antibodies may interfere with this regulatory function [67, 68], potentially amplifying type I IFN signaling; however, this hypothesis requires further investigation [69].

### Mitochondria

Given that MDA5 initiates type I IFN production through a signaling complex on the mitochondrial surface, mitochondrial abnormalities in MDA5-DM have garnered attention. Muscle biopsy specimens from MDA5-DM patients have shown a high frequency of mitochondrial abnormalities and elevated expression of MDA5, MAVS, IRF7, and ISG15 compared to controls [70]. However, overt muscle involvement is relatively infrequent in MDA5-DM, and mitochondrial abnormalities have not been consistently observed in the more commonly affected tissues, such as the lungs and skin, highlighting the need for further investigation [71].

### Fibrosis

Fibrotic changes in the lungs of MDA5-DM patients are an area of growing interest. Cytokine profiling has shown elevated levels of pro-inflammatory cytokines (IL-1, IL-6, TNF-α, IL-18), as well as fibrotic mediators such as IL-8 (CXCL8) and IL-10 [37, 43]. Notably, soluble CXCL16—a chemokine that promotes fibroblast proliferation, migration, and collagen production—is significantly elevated in MDA5-DM and serves as a prognostic marker for RP-ILD [44]. Single-cell transcriptomics of lung tissue have revealed upregulation of fibrosis-related gene signatures [33], and autopsy studies have shown accumulation of CD68⁺, CD163⁺, ferritin-producing M2-like macrophages in the lungs of RP-ILD patients [39, 72, 73].

Further studies suggest that alveolar macrophages and airway epithelial cells produce stromal cell-derived factor-1 (SDF-1), which recruits IL-21–expressing CD4⁺CXCR4⁺ T cells that drive pulmonary fibrosis [74]. These T cells, in turn, stimulate lung fibroblast proliferation and enhance the production of TGF-β, α-SMA, and collagen I. Lung fibroblasts, influenced by IFN-γ and IL-1β, express CX3CL1, which attracts CX3CR1⁺ M2 macrophages, perpetuating the fibrotic cycle [49]. Additionally, IL-21 has been shown to promote the differentiation of pro-fibrotic CD8⁺ T cells that produce IL-13 [75], consistent with findings of increased ISG15⁺CD8⁺ T cells in single-cell analyses [33]. Expression of miR-30b, an anti-fibrotic microRNA secreted by mesenchymal cells, is reduced in MDA5-DM lung biopsies [76], further supporting the fibrotic potential in this disease.

Despite these molecular indications of fibrosis, it is important to note that radiologically and histologically, lung fibrosis in MDA5-DM is generally less pronounced than in anti-ARS antibody syndrome or idiopathic pulmonary fibrosis. Imaging often reveals an organizing pneumonia pattern rather than extensive fibrosis [73], especially in the acute phase, where HRCT typically shows minimal fibrotic changes [77]. Furthermore, serum levels of CXCL10 (IP-10), which may exert anti-fibrotic effects, are elevated in MDA5-DM [37, 43]. Interestingly, in vitro studies have shown that overexpression of MDA5 in human fetal lung fibroblasts suppresses TGF-β–mediated fibrosis [77].

These findings highlight a paradox between the molecular potential for fibrosis and its limited clinical expression, suggesting that the balance between inflammation and tissue repair warrants further exploration.

## Advances of Research in anti-MDA5 Antibody

**Fig. 2:**
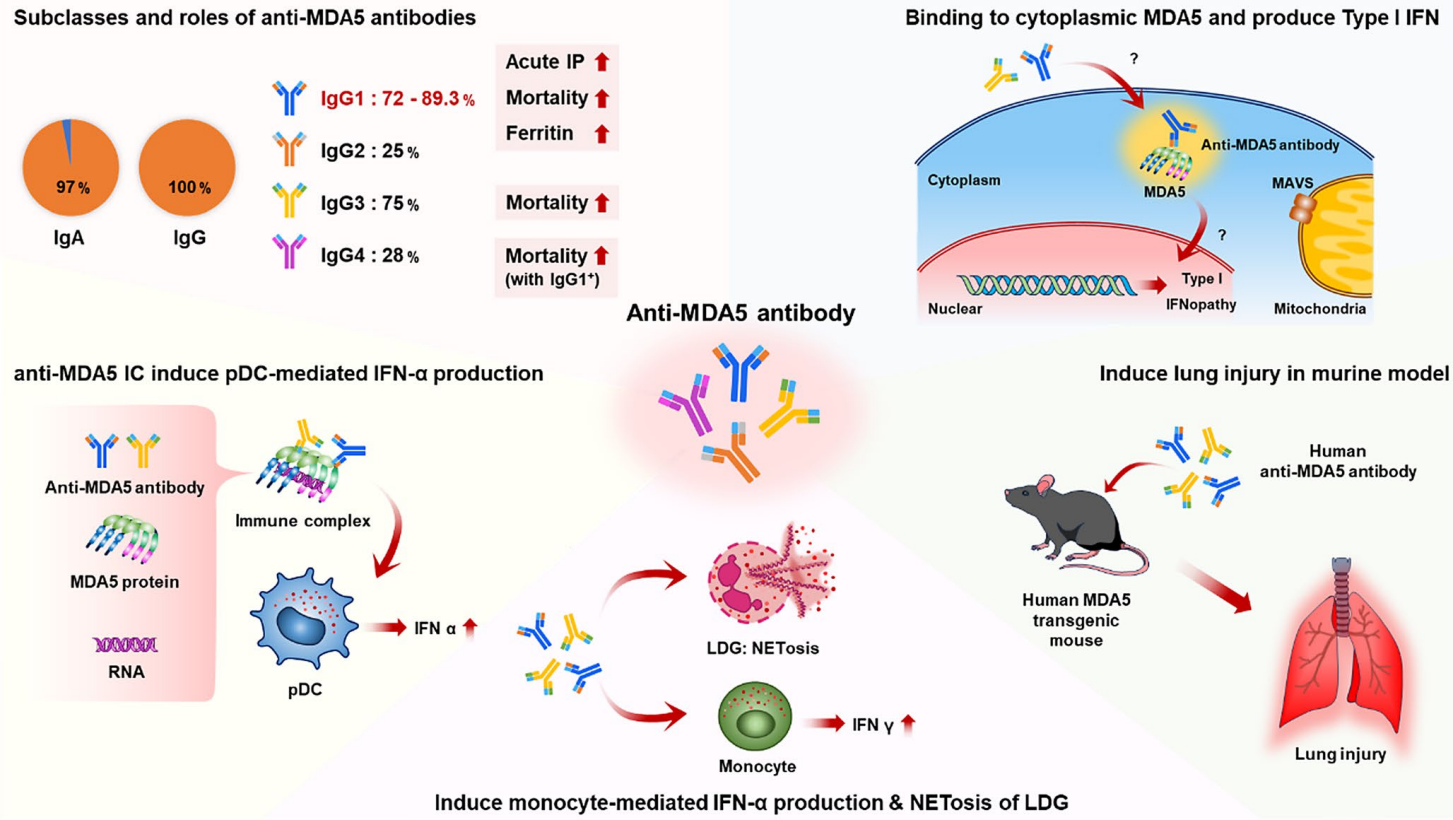
Presumed pathogenesis of anti-MDA5 antibodies. LDG: low-density granulocytes, pDC: plasmacytoid dendritic cells. This figure was originally created by the author using Microsoft PowerPoint (Office 2019)

In recent years, growing attention has been directed toward the potential pathogenic role of anti-MDA5 antibody titers. A series of in vitro studies has provided evidence supporting their pathogenicity (Fig. 2). Moreover, detailed analyses have identified specific Ig subclasses of anti-MDA5 antibodies, revealing ethnic differences in epitope recognition that appear to be associated with distinct clinical phenotypes.

### Pathogenicity of anti-MDA5 Antibodies

Anti-MDA5 antibody titers strongly correlate with the type I IFN signature [78] and are associated with disease activity in MDA5-DM [79]. Furthermore, these antibodies serve as prognostic markers for RP-ILD in CADM patients [7, 19]. In survivors of MDA5-DM, anti-MDA5 antibody titers decline during remission [80], whereas non-survivors often fail to exhibit such reductions [81]. Plasma exchange therapy has shown clinical efficacy in patients with severe disease, and a reduction in anti-MDA5 antibody titers has been observed in survivors following treatment [37]. These findings suggest a potential pathogenic role of anti-MDA5 antibodies and raise the possibility that their removal may contribute to disease amelioration.

In in vitro experiments, the addition of anti-MDA5 antibodies to neutrophils has been shown to induce the formation of neutrophil extracellular traps (NETs) [82]. Furthermore, when monoclonal anti-MDA5 antibodies derived from B cell clones of MDA5-DM patients were added to peripheral blood monocyte cultures, an increase in IFN-γ production was observed [47]. The pathogenic potential of immune complexes containing anti-MDA5 antibodies has also been demonstrated; specifically, artificially generated immune complexes composed of anti-MDA5 antibodies, MDA5 protein, and RNA strongly induced IFN-α production via TLR7 signaling when applied to plasmacytoid dendritic cells [83]. In an in vitro experiment using electroporated muscle cells, the addition of patient-derived purified anti-MDA5 antibodies led to binding of the antibodies to cytoplasmic MDA5, resulting in excessive expression of the type I IFN, IFN-β1, and its downstream interferon-stimulated genes [84]. Although this experiment did not assess pulmonary or vascular cells, and the mechanism by which anti-MDA5 antibodies enter the cytoplasm remains unclear, these findings suggest that intracellular binding of anti-MDA5 antibodies to MDA5 may represent a potential trigger of type I interferonopathy in MDA5-DM.

Experimental models support the pathogenic role of anti-MDA5 antibodies. In mice overexpressing human MDA5 protein, administration of human anti-MDA5 antibodies induced severe pulmonary inflammatory cell infiltration, highlighting their contribution to disease pathogenesis [85]. Interestingly, the administration of polyclonal anti-human MDA5 antibodies to wild-type mice did not induce pneumonia. In contrast, when wild-type mice were immunized with recombinant full-length murine MDA5 protein to induce the production of anti-MDA5 antibodies, followed by intranasal administration of polyinosinic-polycytidylic acid—a synthetic analog of viral double-stranded RNA and an MDA5 agonist, acute lung injury was triggered [86]. These findings suggest that the presence of anti-MDA5 antibodies alone is insufficient to induce lung inflammation; however, in the context of a viral infection that leads to local overexpression and activation of MDA5, the presence of these antibodies may exacerbate pulmonary inflammation.

### Subclasses of anti-MDA5 Antibodies

Studies on the subtypes and subclasses of anti-MDA5 antibodies have also progressed in recent years. The vast majority of anti-MDA5 antibodies are of the IgG isotype (100%); intriguingly, IgA-class anti-MDA5 antibodies are concurrently detected in approximately 97% of patients [87]. However, the clinical significance of these IgA antibodies remains to be determined.

Among IgG subclasses, IgG1 is most commonly associated with poor prognosis [87, 88]. Known for its pro-inflammatory properties, IgG1 promotes the production of inflammatory cytokines such as TNF, IL-1β, and IL-23, and activates the type I IFN response [89]. It also triggers complement activation, potentially contributing to complement-mediated cytotoxicity and immune complex formation.

In addition to IgG1, IgG3 and co-expression of IgG1 and IgG4 are further associated with poor outcomes [87, 88]. These findings suggest that the subtype of anti-MDA5 antibodies, especially the IgG1 subclass, may have a significant impact on disease prognosis. However, further validation is required to clarify their clinical relevance and pathogenic significance.

### Recognition Sites of anti-MDA5 Antibodies

Recent studies have focused on identifying the epitopes of MDA5 recognized by anti-MDA5 antibodies. Research examining the binding affinity of anti-MDA5 antibodies in the serum of Chinese and European patients to various fragmented MDA5 proteins and peptides has revealed that the helicase domain is the primary epitope for these antibodies among the three major domains of MDA5 [90, 91]. Conversely, another study reported distinct patterns of epitope recognition in different populations: anti-MDA5 antibodies in Japanese patients predominantly target fragments of the CARD domain, whereas those in American patients are more likely to recognize fragments of the CTD domain [92].

**Fig. 3:**
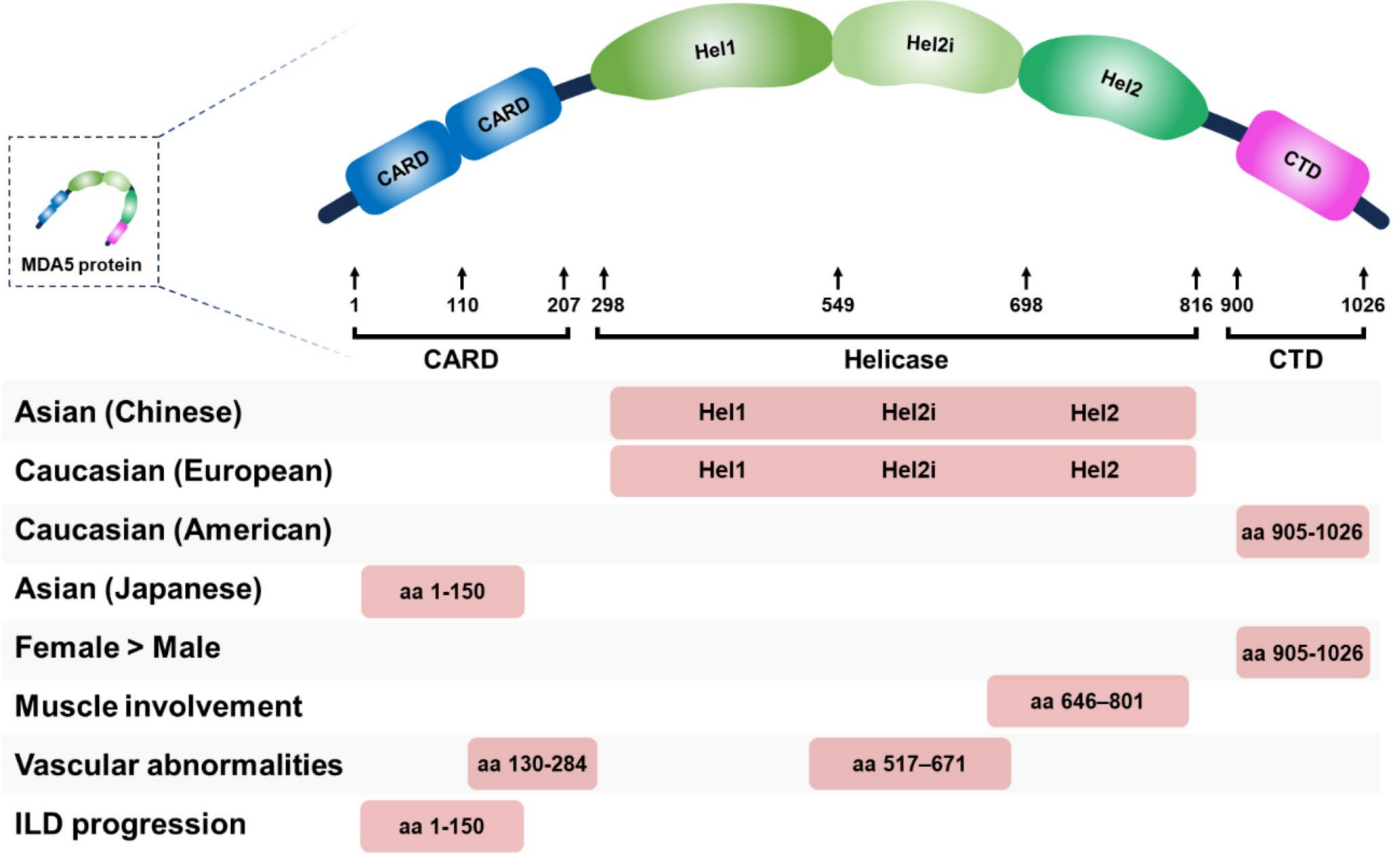
Association between clinical phenotypes and recognition epitopes of anti-MDA5 antibodies. CTD: C-terminal domain, ILD: interstitial lung disease. This figure was originally created by the author using Microsoft PowerPoint (Office 2019)

Additional evidence suggests that differences in epitope recognition by anti-MDA5 antibodies may correlate with clinical phenotypes. For example, anti-MDA5 antibodies recognizing the CTD domain are more frequently detected in women than in men. Patients with muscle or vascular involvement often have antibodies targeting the helicase domain [93]. Additionally, in patients who succumbed to progressive ILD, anti-MDA5 antibodies recognizing fragments of the CARD domain were predominantly observed. These findings suggest that epitope-specific recognition by anti-MDA5 antibodies may contribute to variations in mortality rates and clinical phenotypes across different populations (Fig. 3).

### Mechanisms of anti-MDA5 Antibody Production

While significant progress has been made in understanding the pathogenicity and epitope recognition of anti-MDA5 antibodies, increasing attention is being directed toward elucidating the mechanisms underlying their production.

There has been a case report of an MDA5-DM patient in whom anti-MDA5 antibody levels spontaneously normalized following surgical resection of a pulmonary nodule that had been suspected to be lung cancer during disease relapse [94]. This observation suggests the possibility that the lung may serve as a source of anti-MDA5 antibody production. Additionally, several case reports suggest that the seroconversion of anti-MDA5 antibodies may begin prior to the onset of MDA5-DM [81]. In a retrospective analysis of four patients who developed MDA5-DM following allogeneic hematopoietic stem cell transplantation, three exhibited seropositivity for anti-MDA5 antibodies 43 to 99 days before the onset of ILD [95]. These findings raise the possibility that, similar to anti-CCP antibodies in rheumatoid arthritis, there may be a preclinical stage in MDA5-DM during which anti-MDA5 antibodies are already detectable.

To induce anti-MDA5 antibody production, MDA5, typically localized in the cytoplasm, must be released extracellularly and recognized by antigen-presenting cells. As previously mentioned, MDA5 expression is upregulated in epithelial cells of the lungs and skin, as well as in circulating neutrophils and monocytes in MDA5-DM patients, driven by type I IFN signaling [24–26]. It is hypothesized that cell death of these mesenchymal and immune cells leads to the extracellular release of cytoplasmic MDA5, allowing its recognition by antigen-presenting cells and subsequently triggering autoantibody production.

On the other hand, interestingly, it has been reported that peripheral blood mononuclear cells (PBMCs) from MDA5-DM patients constitutively secrete MDA5 protein [96]. Furthermore, it has been demonstrated that the addition of double-stranded RNA, recognized by MDA5 to PBMCs promotes the extracellular secretion of MDA5. This finding suggests that, for instance, viral infections may not only upregulate MDA5 expression in PBMCs but also promote its secretion in a soluble form. Such processes may contribute not only to systemic type I interferonopathy but also to the production of anti-MDA5 antibodies.

## Treatment of MDA5-DM

### Current Treatment Strategies

Currently, the standard treatment for MDA5-DM involves a combination of high-dose glucocorticoids, calcineurin inhibitors, and high-dose intravenous cyclophosphamide, which has been shown to improve survival outcomes [97]. To suppress the wide array of immune cells, cytokines, and chemokines involved in disease pathogenesis, glucocorticoids are used to broadly inhibit immune responses, calcineurin inhibitors target CD8⁺ and CD4⁺ T cells involved in inflammation and fibrosis, and cyclophosphamide suppresses both T and B cells, collectively reducing disease activity.

Regarding calcineurin inhibitors, an open-label randomized controlled trial comparing tacrolimus and cyclosporine A reported no significant difference in efficacy; however, only 30% of the study participants had MDA5-DM, necessitating cautious interpretation [98]. Moreover, higher tacrolimus blood concentrations than traditionally recommended for idiopathic inflammatory myopathies may be required to prevent relapse in MDA5-DM, and dose adjustments based on CYP3A5 genotype are suggested [99].

A randomized controlled trial evaluating intravenous immune globulin (IVIG) in dermatomyositis demonstrated significant clinical improvements [100], although the proportion of MDA5-DM patients in this study was low. Retrospective observational studies focusing on MDA5-DM have reported that early IVIG administration is effective, similar to other forms of dermatomyositis [101]. However, the potential confounding effects of the initial three-drug combination therapy and uncertainties surrounding optimal IVIG dosing intervals remain challenges.

Therapies aimed at removing pathogenic factors, such as anti-MDA5 antibodies, complement, and immunoglobulin deposits in lung tissues [85], have also been investigated. Polymyxin B-immobilized fiber column hemoperfusion and plasma exchange have shown promise in reducing inflammatory cytokines and antibodies [37, 102, 103]. A multicenter study involving 51 patients did not demonstrate efficacy for plasma exchange [102], while systematic reviews including five retrospective cohort studies, four case-control studies, and two case series have suggested potential benefits [104]. This discrepancy highlights the need for larger-scale studies to establish optimal treatment protocols and determine appropriate durations.

Given the high risk of *Pneumocystis jirovecii* pneumonia (PJP) during intensified immunosuppressive therapy in MDA5-DM [105, 106], prophylaxis with trimethoprim-sulfamethoxazole is likely to improve outcomes [107].

### Emerging Therapeutic Options

For cases refractory to calcineurin inhibitors, mycophenolate mofetil has been reported to improve clinical status [108]. However, azathioprine, methotrexate, and leflunomide have not demonstrated efficacy and are generally not recommended [109].

Although limited data exist on biological agents, there are case reports and case series suggesting the efficacy of tocilizumab [110, 111]. In MDA5-DM, elevated IL-6 levels have been associated with poor prognosis, and activation of the IL-6/STAT3 signaling pathway has been reported in juvenile dermatomyositis, including MDA5 positive cases [112, 113]. However, the clinical use of tocilizumab, an IL-6 receptor inhibitor, remains limited at present in this context.

The use of JAK inhibitors in MDA5-DM has garnered attention, with reports highlighting their efficacy. JAK inhibitors target not only type I IFN signaling, which is central to the pathogenesis of MDA5-DM, but also modulate type II IFN and IL-21 signaling, both of which are implicated in fibrotic processes. These cytokines signal through the JAK-STAT pathway, particularly via the JAK1-STAT1 and JAK3-STAT5 axes [114, 115]. Therefore, JAK inhibition may have the potential to suppress acute-phase inflammation as well as mitigate chronic-phase pulmonary fibrosis in MDA5-DM. Although JAK inhibitors are often administered in combination with conventional triple therapy in cases refractory to standard treatment, meta-analyses suggest that their efficacy is observed regardless of the timing of initiation [116]. Moreover, therapeutic benefits have been reported across different classes of JAK inhibitors, indicating class-independent efficacy [117–120].

B cell depletion therapies, including rituximab, are being explored due to the suggested pathogenic role of anti-MDA5 antibodies and the association of additional autoantibodies, such as anti-Ro, with poor prognosis. Reports indicate that standard-dose (e.g., 1,000 mg biweekly for two doses) and low-dose (e.g., 100 mg weekly for four doses) rituximab regimens have been effective in refractory cases [121–124]. For rituximab-resistant cases, anti-CD38 antibodies such as daratumumab, which target plasma cells, have shown efficacy [125–127]. Importantly, the increase in CD38⁺ CD4⁺ and CD38⁺ CD8⁺ T cells in MDA5-DM, which correlates with poor RP-ILD outcomes, suggests that targeting these specific T cell subsets may provide additional therapeutic benefits [128].

In a small case series, basiliximab, a monoclonal antibody against the IL-2 receptor α-chain (CD25), demonstrated a 75% response rate in four patients with RP-ILD refractory to glucocorticoids and calcineurin inhibitors [129]. However, no additional reports on its efficacy have been published since its initial study in 2014.

A report documented the use of teclistamab, a bispecific antibody targeting B-cell maturation antigen (BCMA) and CD3, in four patients with autoimmune diseases, including MDA5-DM [130]. All four patients demonstrated favorable outcomes, highlighting its potential as a promising new therapeutic option.

Antifibrotic agents have shown efficacy in early-stage disease in some case series [131]. While fibrosis is rare in the acute phase, it becomes more prevalent in chronic cases, suggesting that antifibrotic therapy may benefit select patients [132].

Lung transplantation remains a potential option for end-stage disease. A systematic review of 11 cases reported a 90.1% survival rate without recurrence [133]. However, publication bias and the challenge of determining optimal timing for transplantation should be considered.

**Fig. 4:**
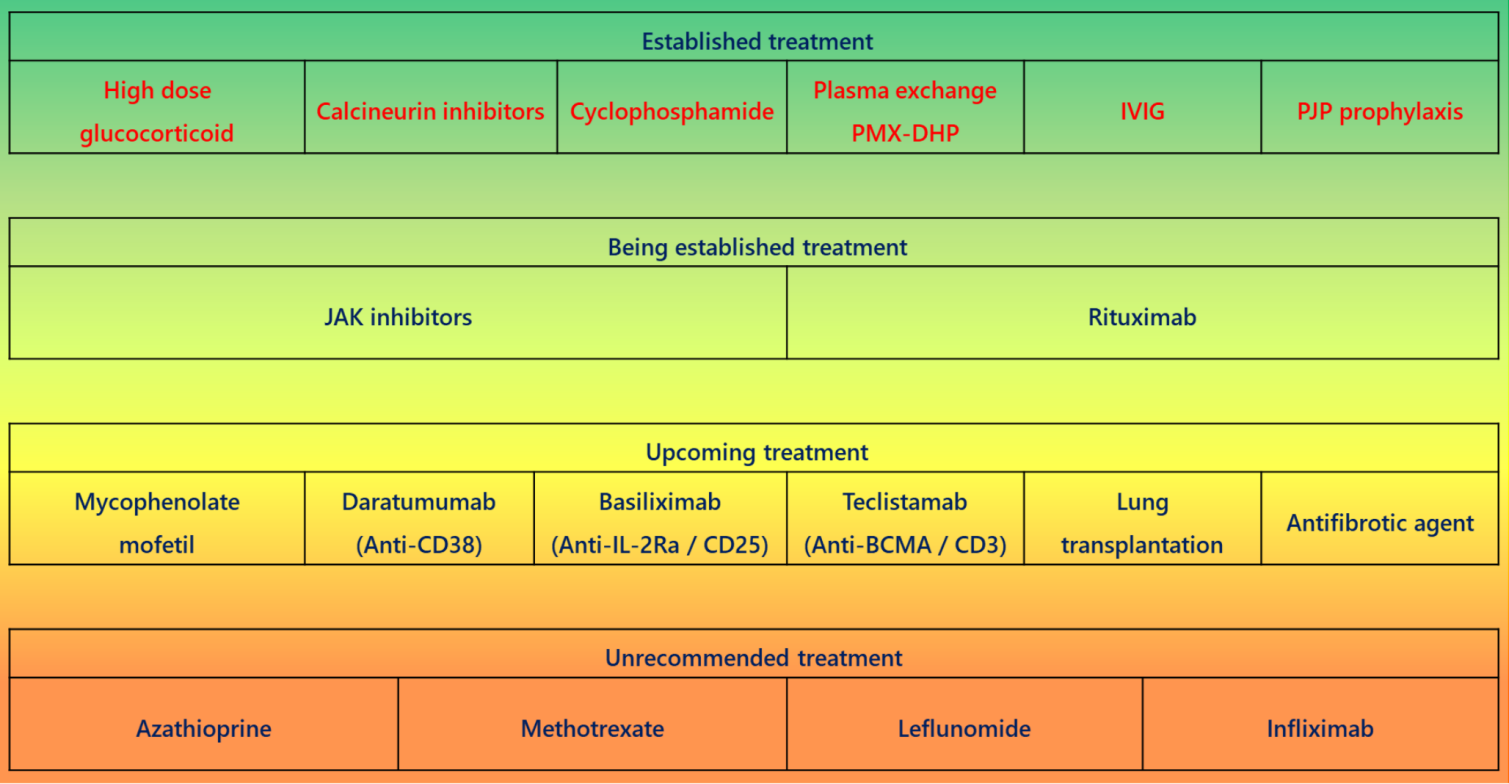
Treatment choice of MDA5-DM. IVIG: Intravenous Immunoglobulin. This figure was originally created by the author using Microsoft PowerPoint (Office 2019)

Current treatment options and future therapeutic prospects are summarized in Fig. 4.

## Conclusion

MDA5-DM predominantly affects Asians, including Japanese individuals, and is characterized by high severity. Recent research has advanced our understanding of various aspects of its pathogenesis, including genetic susceptibility, environmental triggers, immune cell dynamics in affected tissues, the potential positive feedback loop between type I interferonopathy and MDA5 expression, as well as the pathogenicity and subclass profiles of anti-MDA5 antibodies.

In terms of treatment, accumulating evidence has supported the use of novel therapeutic options, such as JAK inhibitors and rituximab in addition to conventional triple-combination therapy. Conversely, emerging reports have highlighted the existence of distinct clinical phenotypes of MDA5-DM with varying degrees of disease severity [134–136]. Going forward, it is imperative to establish accurate phenotypic classifications to identify which patients require aggressive immunosuppressive therapy, ensuring more tailored and effective treatment strategies.

## Electronic Supplementary Material

Below is the link to the electronic supplementary material.

Supplementary Material 1

## Data Availability

No datasets were generated or analysed during the current study.

## References

[1] Nakashima R, Hosono Y, Mimori T. Clinical significance and new detection system of autoantibodies in myositis with interstitial lung disease. Lupus. 2016;25(8):925–33. 27252271 10.1177/0961203316651748

[2] Lin RR, Warp PV, Maderal AD, Elman SA. Assessing time of onset for interstitial lung disease in Anti-MDA5 Antibody-Positive dermatomyositis. JAMA Dermatol. 2024;160(5):575–7. 38598206 10.1001/jamadermatol.2024.0507 PMC11007644

[3] Betteridge Z, Tansley S, Shaddick G, Chinoy H, Cooper RG, New RP, et al. Frequency, mutual exclusivity and clinical associations of myositis autoantibodies in a combined European cohort of idiopathic inflammatory myopathy patients. J Autoimmun. 2019;101:48–55. 30992170 10.1016/j.jaut.2019.04.001 PMC6580360

[4] Nombel A, Fabien N, Coutant F. Dermatomyositis with Anti-MDA5 antibodies: bioclinical features, pathogenesis and emerging therapies. Front Immunol. 2021;12:773352. 34745149 10.3389/fimmu.2021.773352 PMC8564476

[5] Nakashima R, Imura Y, Kobayashi S, Yukawa N, Yoshifuji H, Nojima T, et al. The RIG-I-like receptor IFIH1/MDA5 is a dermatomyositis-specific autoantigen identified by the anti-CADM-140 antibody. Rheumatology (Oxford). 2010;49(3):433–40. 20015976 10.1093/rheumatology/kep375

[6] Muro Y, Sugiura K, Hoshino K, Akiyama M, Tamakoshi K. Epidemiologic study of clinically amyopathic dermatomyositis and anti-melanoma differentiation-associated gene 5 antibodies in central Japan. Arthritis Res Ther. 2011;13(6):R214. 22192091 10.1186/ar3547 PMC3334667

[7] Koga T, Fujikawa K, Horai Y, Okada A, Kawashiri SY, Iwamoto N, et al. The diagnostic utility of anti-melanoma differentiation-associated gene 5 antibody testing for predicting the prognosis of Japanese patients with DM. Rheumatology (Oxford). 2012;51(7):1278–84. 22378718 10.1093/rheumatology/ker518

[8] Sato S, Hirakata M, Kuwana M, Suwa A, Inada S, Mimori T, et al. Autoantibodies to a 140-kd polypeptide, CADM-140, in Japanese patients with clinically amyopathic dermatomyositis. Arthritis Rheum. 2005;52(5):1571–6. 15880816 10.1002/art.21023

[9] Ceribelli A, Fredi M, Taraborelli M, Cavazzana I, Tincani A, Selmi C, et al. Prevalence and clinical significance of anti-MDA5 antibodies in European patients with polymyositis/dermatomyositis. Clin Exp Rheumatol. 2014;32(6):891–7. 25151986

[10] Fujikawa K, Kawakami A, Kaji K, Fujimoto M, Kawashiri S, Iwamoto N, et al. Association of distinct clinical subsets with myositis-specific autoantibodies towards anti-155/140-kDa polypeptides, anti-140-kDa polypeptides, and anti-aminoacyl tRNA synthetases in Japanese patients with dermatomyositis: a single-centre, cross-sectional study. Scand J Rheumatol. 2009;38(4):263–7. 19444719 10.1080/03009740802687455

[11] Fiorentino D, Chung L, Zwerner J, Rosen A, Casciola-Rosen L. The mucocutaneous and systemic phenotype of dermatomyositis patients with antibodies to MDA5 (CADM-140): a retrospective study. J Am Acad Dermatol. 2011;65(1):25–34. 21531040 10.1016/j.jaad.2010.09.016 PMC3167687

[12] Labrador-Horrillo M, Martinez MA, Selva-O’Callaghan A, Trallero-Araguas E, Balada E, Vilardell-Tarres M, et al. Anti-MDA5 antibodies in a large mediterranean population of adults with dermatomyositis. J Immunol Res. 2014;2014:290797. 24741583 10.1155/2014/290797 PMC3987881

[13] Gono T, Kawaguchi Y, Kuwana M, Sugiura T, Furuya T, Takagi K, et al. Brief report: association of HLA-DRB1*0101/*0405 with susceptibility to anti-melanoma differentiation-associated gene 5 antibody-positive dermatomyositis in the Japanese population. Arthritis Rheum. 2012;64(11):3736–40. 22886382 10.1002/art.34657

[14] Rothwell S, Chinoy H, Lamb JA, Miller FW, Rider LG, Wedderburn LR, et al. Focused HLA analysis in Caucasians with myositis identifies significant associations with autoantibody subgroups. Ann Rheum Dis. 2019;78(7):996–1002. 31138531 10.1136/annrheumdis-2019-215046 PMC6585280

[15] Kochi Y, Kamatani Y, Kondo Y, Suzuki A, Kawakami E, Hiwa R, et al. Splicing variant of WDFY4 augments MDA5 signalling and the risk of clinically amyopathic dermatomyositis. Ann Rheum Dis. 2018;77(4):602–11. 29331962 10.1136/annrheumdis-2017-212149

[16] Guo L, Zhang X, Pu W, Zhao J, Wang K, Zhang D, et al. WDFY4 polymorphisms in Chinese patients with anti-MDA5 dermatomyositis is associated with rapid progressive interstitial lung disease. Rheumatology (Oxford). 2023;62(6):2320–4. 36637178 10.1093/rheumatology/kead006

[17] Theisen DJ, Davidson JT, Briseño CG, Gargaro M, Lauron EJ, Wang Q, et al. WDFY4 is required for cross-presentation in response to viral and tumor antigens. Science. 2018;362(6415):694–9. 30409884 10.1126/science.aat5030 PMC6655551

[18] Li Y, Li J, Yuan Q, Bian X, Long F, Duan R, et al. Deficiency in WDFY4 reduces the number of CD8 + T cells via reactive oxygen species-induced apoptosis. Mol Immunol. 2021;139:131–8. 34482201 10.1016/j.molimm.2021.08.022

[19] Chen Z, Cao M, Plana MN, Liang J, Cai H, Kuwana M, et al. Utility of anti-melanoma differentiation-associated gene 5 antibody measurement in identifying patients with dermatomyositis and a high risk for developing rapidly progressive interstitial lung disease: a review of the literature and a meta-analysis. Arthritis Care Res (Hoboken). 2013;65(8):1316–24. 23908005 10.1002/acr.21985

[20] Nishina N, Sato S, Masui K, Gono T, Kuwana M. Seasonal and residential clustering at disease onset of anti-MDA5-associated interstitial lung disease. RMD Open. 2020;6(2):e001202. 32506053 10.1136/rmdopen-2020-001202 PMC7299503

[21] Toquet S, Granger B, Uzunhan Y, Mariampillai K, Nunes H, Benveniste O, et al. The seasonality of dermatomyositis associated with anti-MDA5 antibody: an argument for a respiratory viral trigger. Autoimmun Rev. 2021;20(4):102788. 33609802 10.1016/j.autrev.2021.102788

[22] Palterer B, Mazzoni A, Infantino M, Semeraro R, Manfredi M, Pesce G, et al. Seasonal patterns of myositis-specific and myositis-associated autoantibodies in Italy: seasonal patterns of myositis autoantibodies. Immunol Lett. 2024;272:106966. 39681209 10.1016/j.imlet.2024.106966

[23] So H, So J, Lam TTO, Wong VTL, Ho R, Li WL, et al. Seasonal effect on disease onset and presentation in Anti-MDA5 positive dermatomyositis. Front Med (Lausanne). 2022;9:837024. 35187011 10.3389/fmed.2022.837024 PMC8854504

[24] Kang Dchul, Gopalkrishnan RV, Wu Q, Jankowsky E, Pyle AM, Fisher PB. mda-5: an interferon-inducible putative RNA helicase with double-stranded RNA-dependent ATPase activity and melanoma growth-suppressive properties. Proc Natl Acad Sci U S A. 2002;99(2):637–42. 11805321 10.1073/pnas.022637199 PMC117358

[25] Kang DC, Gopalkrishnan RV, Lin L, Randolph A, Valerie K, Pestka S, et al. Expression analysis and genomic characterization of human melanoma differentiation associated gene-5, mda-5: a novel type I interferon-responsive apoptosis-inducing gene. Oncogene. 2004;23(9):1789–800. 14676839 10.1038/sj.onc.1207300

[26] Zahn S, Barchet W, Rehkämper C, Hornung T, Bieber T, Tüting T, et al. Enhanced skin expression of melanoma differentiation-associated gene 5 (MDA5) in dermatomyositis and related autoimmune diseases. J Am Acad Dermatol. 2011;64(5):988–9. 21496705 10.1016/j.jaad.2010.08.004

[27] Gono T, Okazaki Y, Kuwana M. Antiviral Proinflammatory phenotype of monocytes in anti-MDA5 antibody-associated interstitial lung disease. Rheumatology (Oxford). 2022;61(2):806–14. 33890985 10.1093/rheumatology/keab371

[28] Liu Y, Feng S, Liu X, Tang Y, Li X, Luo C, et al. IFN-beta and EIF2AK2 are potential biomarkers for interstitial lung disease in anti-MDA5 positive dermatomyositis. Rheumatology (Oxford). 2023;62(11):3724–31. 36912714 10.1093/rheumatology/kead117

[29] Jayaraman S, Tiniakou E, Morgenlander WR, Na M, Christopher-Stine L, Larman HB. Comprehensive enteroviral serology links infection and Anti-Melanoma Differentiation-Associated protein 5 dermatomyositis. ACR Open Rheumatol; 2024. 10.1002/acr2.11752 PMC11694254 39509140

[30] Castellini C, Scotti C, Navarini L, Fu Q, Qian J, Giacomelli R, et al. The evaluation of type I interferon score in dermatomyositis, a systematic review and a meta-analysis. Autoimmun Rev. 2024;23(12):103686. 39521363 10.1016/j.autrev.2024.103686

[31] Zhang SH, Zhao Y, Xie QB, Jiang Y, Wu YK, Yan B. Aberrant activation of the type I interferon system May contribute to the pathogenesis of anti-melanoma differentiation-associated gene 5 dermatomyositis. Br J Dermatol. 2019;180(5):1090–8. 29947075 10.1111/bjd.16917

[32] Cassius C, Amode R, Delord M, Battistella M, Poirot J, How-Kit A, et al. MDA5 + Dermatomyositis is associated with stronger skin type I interferon transcriptomic signature with upregulation of IFN-κ transcript. J Invest Dermatol. 2020;140(6):1276–e12797. 31955963 10.1016/j.jid.2019.10.020

[33] Ye Y, Chen Z, Jiang S, Jia F, Li T, Lu X, et al. Single-cell profiling reveals distinct adaptive immune hallmarks in MDA5 + dermatomyositis with therapeutic implications. Nat Commun. 2022;13(1):6458. 36309526 10.1038/s41467-022-34145-4 PMC9617246

[34] Ono N, Kai K, Maruyama A, Sakai M, Sadanaga Y, Koarada S, et al. The relationship between type 1 IFN and vasculopathy in anti-MDA5 antibody-positive dermatomyositis patients. Rheumatology (Oxford). 2019;58(5):786–91. 30541137 10.1093/rheumatology/key386

[35] Qian J, Li R, Chen Z, Cao Z, Lu L, Fu Q. Type I interferon score is associated with the severity and poor prognosis in anti-MDA5 antibody-positive dermatomyositis patients. Front Immunol. 2023;14:1151695. 37006269 10.3389/fimmu.2023.1151695 PMC10063972

[36] Funabiki M, Kato H, Miyachi Y, Toki H, Motegi H, Inoue M, et al. Autoimmune disorders associated with gain of function of the intracellular sensor MDA5. Immunity. 2014;40(2):199–212. 24530055 10.1016/j.immuni.2013.12.014

[37] Shirakashi M, Nakashima R, Tsuji H, Tanizawa K, Handa T, Hosono Y, et al. Efficacy of plasma exchange in anti-MDA5-positive dermatomyositis with interstitial lung disease under combined immunosuppressive treatment. Rheumatology (Oxford). 2020;59(11):3284–92. 32276271 10.1093/rheumatology/keaa123

[38] Fujisawa T, Hozumi H, Yasui H, Suzuki Y, Karayama M, Furuhashi K, et al. Clinical significance of serum Chitotriosidase level in Anti-MDA5 Antibody-positive Dermatomyositis-associated interstitial lung disease. J Rheumatol. 2019;46(8):935–42. 31092718 10.3899/jrheum.180825

[39] Enomoto Y, Suzuki Y, Hozumi H, Mori K, Kono M, Karayama M, et al. Clinical significance of soluble CD163 in polymyositis-related or dermatomyositis-related interstitial lung disease. Arthritis Res Ther. 2017;19(1):9. 28103926 10.1186/s13075-016-1214-8 PMC5248519

[40] Liang L, Zhang YM, Shen YW, Song AP, Li WL, Ye LF, et al. Aberrantly expressed Galectin-9 is involved in the Immunopathogenesis of Anti-MDA5-Positive Dermatomyositis-Associated interstitial lung disease. Front Cell Dev Biol. 2021;9:628128. 33842457 10.3389/fcell.2021.628128 PMC8027128

[41] Kokuzawa A, Nakamura J, Kamata Y, Sato K. Potential role of type I interferon/IP-10 axis in the pathogenesis of anti-MDA5 antibody-positive dermatomyositis. Clin Exp Rheumatol. 2023;41(2):275–84. 36622131 10.55563/clinexprheumatol/em67zx

[42] Kuzumi A, Fukasawa T, Yamashita T, Matsuda KM, Kotani H, Yoshizaki-Ogawa A et al. Serum interleukin-34 levels in dermatomyositis: a potential biomarker for anti-MDA5-antibody-associated interstitial lung disease. Rheumatology (Oxford). 2024;keae313. 10.1093/rheumatology/keae313 38830088

[43] Gono T, Kaneko H, Kawaguchi Y, Hanaoka M, Kataoka S, Kuwana M, et al. Cytokine profiles in polymyositis and dermatomyositis complicated by rapidly progressive or chronic interstitial lung disease. Rheumatology (Oxford). 2014;53(12):2196–203. 24970922 10.1093/rheumatology/keu258

[44] Thuner J, Coutant F. IFN-γ: an overlooked cytokine in dermatomyositis with anti-MDA5 antibodies. Autoimmun Rev. 2023;22(10):103420. 37625674 10.1016/j.autrev.2023.103420

[45] Fukada A, Fujisawa T, Hozumi H, Koda K, Akamatsu T, Oyama Y, et al. Prognostic role of Interferon-λ3 in Anti-Melanoma Differentiation-Associated gene 5-Positive Dermatomyositis-Associated interstitial lung disease. Arthritis Rheumatol. 2024;76(5):796–805. 38146102 10.1002/art.42785

[46] Ishikawa Y, Iwata S, Hanami K, Nawata A, Zhang M, Yamagata K, et al. Relevance of interferon-gamma in pathogenesis of life-threatening rapidly progressive interstitial lung disease in patients with dermatomyositis. Arthritis Res Ther. 2018;20(1):240. 30367666 10.1186/s13075-018-1737-2 PMC6235206

[47] Coutant F, Bachet R, Pin JJ, Alonzo M, Miossec P. Monoclonal antibodies from B cells of patients with anti-MDA5 antibody-positive dermatomyositis directly stimulate interferon gamma production. J Autoimmun. 2022;130:102831. 35436746 10.1016/j.jaut.2022.102831

[48] Ishida Y, Kimura A, Nosaka M, Kuninaka Y, Hemmi H, Sasaki I, et al. Essential involvement of the CX3CL1-CX3CR1 axis in bleomycin-induced pulmonary fibrosis via regulation of fibrocyte and M2 macrophage migration. Sci Rep. 2017;7(1):16833. 29203799 10.1038/s41598-017-17007-8 PMC5714949

[49] Isozaki T, Otsuka K, Sato M, Takahashi R, Wakabayashi K, Yajima N, et al. Synergistic induction of CX3CL1 by interleukin-1β and interferon-γ in human lung fibroblasts: involvement of signal transducer and activator of transcription 1 signaling pathways. Transl Res. 2011;157(2):64–70. 21256458 10.1016/j.trsl.2010.11.007

[50] Tsoi LC, Gharaee-Kermani M, Berthier CC, Nault T, Hile GA, Estadt SN, et al. IL18-containing 5-gene signature distinguishes histologically identical dermatomyositis and lupus erythematosus skin lesions. JCI Insight. 2020;5(16):e139558. 32644977 10.1172/jci.insight.139558 PMC7455118

[51] Wen J, Zhou M, Lai Y, Zhuang L, Shi J, Lin Z et al. Serum level of IFN-λ is elevated in idiopathic inflammatory myopathies. Clin Rheumatol. 2024. 10.1007/s10067-024-07227-5 39579267

[52] Huang W, Ren F, Luo L, Zhou J, Huang D, Pan Z, et al. The characteristics of lymphocytes in patients positive for anti-MDA5 antibodies in interstitial lung disease. Rheumatology (Oxford). 2020;59(12):3886–91. 32535634 10.1093/rheumatology/keaa266

[53] Chen F, Wang D, Shu X, Nakashima R, Wang G. Anti-MDA5 antibody is associated with A/SIP and decreased T cells in peripheral blood and predicts poor prognosis of ILD in Chinese patients with dermatomyositis. Rheumatol Int. 2012;32(12):3909–15. 22198664 10.1007/s00296-011-2323-y

[54] Jin Q, Fu L, Yang H, Chen X, Lin S, Huang Z, et al. Peripheral lymphocyte count defines the clinical phenotypes and prognosis in patients with anti-MDA5-positive dermatomyositis. J Intern Med. 2023;293(4):494–507. 36682032 10.1111/joim.13607

[55] Tian Y, He P, Ren L, Xin H, Xi B, Zou R et al. Dynamic change of lymphocytes associated with short-term prognosis in anti-MDA5-positive dermatomyositis with interstitial lung disease: a multicenter retrospective study. Clin Rheumatol. 2024. 10.1007/s10067-024-07110-3 PMC11489275 39292419

[56] Shi J, Pei X, Peng J, Wu C, Lv Y, Wang X, et al. Monocyte-macrophage dynamics as key in disparate lung and peripheral immune responses in severe anti-melanoma differentiation-associated gene 5-positive dermatomyositis-related interstitial lung disease. Clin Transl Med. 2025;15(2):e70226. 39902678 10.1002/ctm2.70226 PMC11791760

[57] Zhang L, Xia Q, Li W, Peng Q, Yang H, Lu X, et al. The RIG-I pathway is involved in peripheral T cell lymphopenia in patients with dermatomyositis. Arthritis Res Ther. 2019;21(1):131. 31142372 10.1186/s13075-019-1905-z PMC6542107

[58] Matsushita T, Kobayashi T, Kano M, Hamaguchi Y, Takehara K. Elevated serum B-cell activating factor levels in patients with dermatomyositis: association with interstitial lung disease. J Dermatol. 2019;46(12):1190–6. 31631384 10.1111/1346-8138.15117

[59] Shi Y, You H, Liu C, Qiu Y, Lv C, Zhu Y, et al. Elevated serum B-cell activator factor levels predict rapid progressive interstitial lung disease in anti-melanoma differentiation associated protein 5 antibody positive dermatomyositis. Orphanet J Rare Dis. 2024;19(1):170. 38637830 10.1186/s13023-024-03153-6 PMC11027411

[60] Wang Y, Zhu L, Ju B, Luo J, Li Q, Lv X, et al. Alterations of peripheral blood B cell subsets in Chinese patients with adult idiopathic inflammatory myopathies. Clin Exp Rheumatol. 2022;40(2):260–6. 34905483 10.55563/clinexprheumatol/ohsmuj

[61] Sugimori Y, Iwasaki Y, Takeshima Y, Okubo M, Kobayashi S, Hatano H, et al. Transcriptome profiling of immune cell types in peripheral blood reveals common and specific pathways involved in the pathogenesis of Myositis-Specific Antibody-Positive inflammatory myopathies. ACR Open Rheumatol. 2023;5(2):93–102. 36651871 10.1002/acr2.11521 PMC9926062

[62] Lv C, You H, Xu L, Wang L, Yuan F, Li J, et al. Coexistence of Anti-Ro52 antibodies in Anti-MDA5 Antibody-Positive dermatomyositis is highly associated with rapidly progressive interstitial lung disease and mortality risk. J Rheumatol. 2023;50(2):219–26. 35705235 10.3899/jrheum.220139

[63] Xu A, Ye Y, Fu Q, Lian X, Chen S, Guo Q, et al. Prognostic values of anti-Ro52 antibodies in anti-MDA5-positive clinically amyopathic dermatomyositis associated with interstitial lung disease. Rheumatology (Oxford). 2021;60(7):3343–51. 33331866 10.1093/rheumatology/keaa786

[64] Gui X, Shenyun S, Ding H, Wang R, Tong J, Yu M, et al. Anti-Ro52 antibodies are associated with the prognosis of adult idiopathic inflammatory myopathy-associated interstitial lung disease. Rheumatology (Oxford). 2022;61(11):4570–8. 35148366 10.1093/rheumatology/keac090

[65] Hosono Y, Nakashima R, Serada S, Murakami K, Imura Y, Yoshifuji H, et al. Splicing factor proline/glutamine-rich is a novel autoantigen of dermatomyositis and associated with anti-melanoma differentiation-associated gene 5 antibody. J Autoimmun. 2017;77:116–22. 27919567 10.1016/j.jaut.2016.11.006

[66] Decker P, Moulinet T, Pontille F, Cravat M, De Carvalho Bittencourt M, Jaussaud R. An updated review of anti-Ro52 (TRIM21) antibodies impact in connective tissue diseases clinical management. Autoimmun Rev. 2022;21(3):103013. 34896652 10.1016/j.autrev.2021.103013

[67] Higgs R, Ní Gabhann J, Ben Larbi N, Breen EP, Fitzgerald KA, Jefferies CA. The E3 ubiquitin ligase Ro52 negatively regulates IFN-beta production post-pathogen recognition by polyubiquitin-mediated degradation of IRF3. J Immunol. 2008;181(3):1780–6. 18641315 10.4049/jimmunol.181.3.1780 PMC2824853

[68] Higgs R, Lazzari E, Wynne C, Ní Gabhann J, Espinosa A, Wahren-Herlenius M, et al. Self protection from anti-viral responses–Ro52 promotes degradation of the transcription factor IRF7 downstream of the viral Toll-Like receptors. PLoS ONE. 2010;5(7):e11776. 20668674 10.1371/journal.pone.0011776 PMC2909902

[69] Espinosa A, Hennig J, Ambrosi A, Anandapadmanaban M, Abelius MS, Sheng Y, et al. Anti-Ro52 autoantibodies from patients with Sjögren’s syndrome inhibit the Ro52 E3 ligase activity by blocking the E3/E2 interface. J Biol Chem. 2011;286(42):36478–91. 21862588 10.1074/jbc.M111.241786 PMC3196112

[70] Jiang Y, Liu Y, Zhao Y, Zheng Y, Yu M, Deng J, et al. Mitochondrial morphology and MAVS-IFN1 signaling pathway in muscles of anti-MDA5 dermatomyositis. Ann Clin Transl Neurol. 2021;8(3):677–86. 33576578 10.1002/acn3.51311 PMC7951095

[71] Li C, Han Y, Li X, Zhang H, Yao Z, Zhou J, et al. Soluble CXCL16 is a prognostic biomarker associated with rapidly progressive interstitial lung disease complicated with dermatomyositis. Semin Arthritis Rheum. 2024;67:152483. 38843569 10.1016/j.semarthrit.2024.152483

[72] Gono T, Miyake K, Kawaguchi Y, Kaneko H, Shinozaki M, Yamanaka H. Hyperferritinaemia and macrophage activation in a patient with interstitial lung disease with clinically amyopathic DM. Rheumatology (Oxford). 2012;51(7):1336–8. 22361226 10.1093/rheumatology/kes012

[73] Zuo Y, Ye L, Liu M, Li S, Liu W, Chen F, et al. Clinical significance of radiological patterns of HRCT and their association with macrophage activation in dermatomyositis. Rheumatology (Oxford). 2020;59(10):2829–37. 32065646 10.1093/rheumatology/keaa034

[74] Wang K, Zhao J, Chen Z, Li T, Tan X, Zheng Y, et al. CD4 + CXCR4 + T cells as a novel prognostic biomarker in patients with idiopathic inflammatory myopathy-associated interstitial lung disease. Rheumatology (Oxford). 2019;58(3):511–21. 30508148 10.1093/rheumatology/key341

[75] Brodeur TY, Robidoux TE, Weinstein JS, Craft J, Swain SL, Marshak-Rothstein A. IL-21 promotes pulmonary fibrosis through the induction of profibrotic CD8 + T cells. J Immunol. 2015;195(11):5251–60. 26519529 10.4049/jimmunol.1500777 PMC4655158

[76] Zhu L, Xu Y, Wang J, Zhang Y, Zhou J, Wu H. Mesenchymal stem cells-derived exosomes carrying microRNA-30b confer protection against pulmonary fibrosis by downregulating Runx1 via Spred2. Mol Genet Genomics. 2024;299(1):33. 38478174 10.1007/s00438-024-02116-7

[77] Shen N, Zhou X, Jin X, Lu C, Hu X, Zhang Y, et al. MDA5 expression is associated with TGF-β-induced fibrosis: potential mechanism of interstitial lung disease in anti-MDA5 dermatomyositis. Rheumatology (Oxford). 2022;62(1):373–83. 35412608 10.1093/rheumatology/keac234

[78] Wang Y, Jia H, Li W, Liu H, Tu M, Li J, et al. Transcriptomic profiling and longitudinal study reveal the relationship of anti-MDA5 titer and type I IFN signature in MDA5 + dermatomyositis. Front Immunol. 2023;14:1249844. 37701443 10.3389/fimmu.2023.1249844 PMC10494241

[79] Matsushita T, Mizumaki K, Kano M, Yagi N, Tennichi M, Takeuchi A, et al. Antimelanoma differentiation-associated protein 5 antibody level is a novel tool for monitoring disease activity in rapidly progressive interstitial lung disease with dermatomyositis. Br J Dermatol. 2017;176(2):395–402. 27452897 10.1111/bjd.14882

[80] Muro Y, Sugiura K, Hoshino K, Akiyama M. Disappearance of anti-MDA-5 autoantibodies in clinically amyopathic DM/interstitial lung disease during disease remission. Rheumatology (Oxford). 2012;51(5):800–4. 22210662 10.1093/rheumatology/ker408

[81] Abe Y, Matsushita M, Tada K, Yamaji K, Takasaki Y, Tamura N. Clinical characteristics and change in the antibody titres of patients with anti-MDA5 antibody-positive inflammatory myositis. Rheumatology (Oxford). 2017;56(9):1492–7. 28499006 10.1093/rheumatology/kex188

[82] Seto N, Torres-Ruiz JJ, Carmona-Rivera C, Pinal-Fernandez I, Pak K, Purmalek MM, et al. Neutrophil dysregulation is pathogenic in idiopathic inflammatory myopathies. JCI Insight. 2020;5(3):e134189. 31945019 10.1172/jci.insight.134189 PMC7098779

[83] Wang K, Zhao J, Wu W, Xu W, Sun S, Chen Z, et al. RNA-Containing immune complexes formed by Anti-Melanoma differentiation associated gene 5 autoantibody are potent inducers of IFN-α. Front Immunol. 2021;12:743704. 34721411 10.3389/fimmu.2021.743704 PMC8554111

[84] Pinal-Fernandez I, Muñoz-Braceras S, Casal-Dominguez M, Pak K, Torres-Ruiz J, Musai J et al. Pathological autoantibody internalisation in myositis. Ann Rheum Dis. 2024;ard-2024-225773. 10.1136/ard-2024-225773 PMC11493519 38902010

[85] Zaizen Y, Okamoto M, Azuma K, Fukuoka J, Hozumi H, Sakamoto N, et al. Enhanced immune complex formation in the lungs of patients with dermatomyositis. Respir Res. 2023;24(1):86. 36934274 10.1186/s12931-023-02362-0 PMC10024827

[86] Ichimura Y, Konishi R, Shobo M, Tanaka R, Kubota N, Kayama H, et al. Autoimmunity against melanoma differentiation-associated gene 5 induces interstitial lung disease mimicking dermatomyositis in mice. Proc Natl Acad Sci U S A. 2024;121(16):e2313070121. 38588434 10.1073/pnas.2313070121 PMC11032490

[87] Chen M, Zhao Q, Diao L, Xue K, Ruan Y, Xue F, et al. Distribution of anti-melanoma differentiation associated gene 5 (MDA5) IgG subclasses in MDA5 + dermatomyositis. Rheumatology (Oxford). 2021;61(1):430–9. 33742662 10.1093/rheumatology/keab268

[88] Xu YT, Zhang YM, Yang HX, Ye LF, Chen F, Lu X, et al. Evaluation and validation of the prognostic value of anti-MDA5 IgG subclasses in dermatomyositis-associated interstitial lung disease. Rheumatology (Oxford). 2022;62(1):397–406. 35412602 10.1093/rheumatology/keac229

[89] Hoepel W, Allahverdiyeva S, Harbiye H, de Taeye SW, van der Ham AJ, de Boer L, et al. IgG subclasses shape cytokine responses by human myeloid immune cells through differential metabolic reprogramming. J Immunol. 2020;205(12):3400–7. 33188071 10.4049/jimmunol.2000263

[90] Mo Y, Ye Y, Peng L, Sun X, Zhong X, Wu R. The central helicase domain holds the major conformational epitopes of melanoma differentiation-associated gene 5 autoantibodies. Rheumatology (Oxford). 2024;63(5):1456–65. 37551942 10.1093/rheumatology/kead397 PMC11065446

[91] Van Gompel E, Demirdal D, Fernandes-Cerqueira C, Horuluoglu B, Galindo-Feria A, Wigren E, et al. Autoantibodies against the melanoma differentiation-associated protein 5 in patients with dermatomyositis target the helicase domains. Rheumatology (Oxford). 2024;63(5):1466–73. 37572295 10.1093/rheumatology/kead400 PMC11065437

[92] Yamaguchi K, Poland P, Zhu L, Moghadam-Kia S, Aggarwal R, Maeno T et al. Comparative B cell epitope profiling in Japanese and North American cohorts of MDA5 + dermatomyositis reveals a direct association between immune repertoire and pulmonary mortality. Rheumatology (Oxford). 2024;keae466. 10.1093/rheumatology/keae466 39186037

[93] Yamaguchi K, Poland P, Bijoy George T, Saygin D, Moghadam-Kia S, Aggarwal R, et al. Correlation between B-cell epitope profile and clinical features of anti-MDA5 antibody-positive dermatomyositis. Rheumatology (Oxford). 2024;63(7):2016–23. 37815819 10.1093/rheumatology/kead550

[94] Hara R, Watanabe S, Terada N, Kase K, Muto A, Hamaguchi Y et al. The lung as a site for the generation of anti-MDA5 antibody in clinically amyopathic dermatomyositis. Rheumatology (Oxford). 2024;keae314. 10.1093/rheumatology/keae314 38833678

[95] Tamaki M, Matsumi S, Nakasone H, Nakamura Y, Kawamura M, Kawamura S, et al. Interstitial lung disease with anti-melanoma differentiation-associated gene 5 antibody after allogeneic hematopoietic stem cell transplantation. Bone Marrow Transpl. 2022;57(9):1382–8. 10.1038/s41409-022-01730-6 PMC9166177 35661835

[96] Okamoto M, Zaizen Y, Kaieda S, Nouno T, Koga T, Matama G, et al. Soluble form of the MDA5 protein in human Sera. Heliyon. 2024;10(11):e31727. 38845920 10.1016/j.heliyon.2024.e31727 PMC11153190

[97] Tsuji H, Nakashima R, Hosono Y, Imura Y, Yagita M, Yoshifuji H, et al. Multicenter prospective study of the efficacy and safety of combined immunosuppressive therapy with High-Dose glucocorticoid, tacrolimus, and cyclophosphamide in interstitial lung diseases accompanied by Anti-Melanoma Differentiation-Associated gene 5-Positive dermatomyositis. Arthritis Rheumatol. 2020;72(3):488–98. 31524333 10.1002/art.41105

[98] Fujisawa T, Hozumi H, Kamiya Y, Kaida Y, Akamatsu T, Kusagaya H, et al. Prednisolone and tacrolimus versus prednisolone and cyclosporin A to treat polymyositis/dermatomyositis-associated ILD: A randomized, open-label trial. Respirology. 2021;26(4):370–7. 33179395 10.1111/resp.13978

[99] Tian X, Liu L, Liu S, Yang J. Tacrolimus personalized therapy based on CYP3A5 genotype in Chinese patients with idiopathic inflammatory myopathies. Rheumatology (Oxford). 2024;keae316. 10.1093/rheumatology/keae316 38889292

[100] Aggarwal R, Charles-Schoeman C, Schessl J, Bata-Csörgő Z, Dimachkie MM, Griger Z, et al. Trial of intravenous immune Globulin in dermatomyositis. N Engl J Med. 2022;387(14):1264–78. 36198179 10.1056/NEJMoa2117912

[101] Wang LM, Yang QH, Zhang L, Liu SY, Zhang PP, Zhang X, et al. Intravenous Immunoglobulin for interstitial lung diseases of anti-melanoma differentiation-associated gene 5-positive dermatomyositis. Rheumatology (Oxford). 2022;61(9):3704–10. 34940809 10.1093/rheumatology/keab928

[102] Bay P, de Chambrun MP, Rothstein V, Mahevas M, De Prost N, Roux A, et al. Efficacy of plasma exchange in patients with anti-MDA5 rapidly progressive interstitial lung disease. J Autoimmun. 2022;133:102941. 36323067 10.1016/j.jaut.2022.102941

[103] Saito T, Mizobuchi M, Miwa Y, Sugiyama M, Mima Y, Iida A, et al. Anti-MDA-5 antibody-positive clinically amyopathic dermatomyositis with rapidly progressive interstitial lung disease treated with therapeutic plasma exchange: A case series. J Clin Apher. 2021;36(1):196–205. 32823371 10.1002/jca.21833

[104] Yang Y, Yang YT, Huo RX, Meng DL, Huang XX, Lin JY. Short-term efficiency of plasma exchange in combination with immunosuppressants and/or biologics in the treatment of idiopathic inflammatory myopathy with rapidly progressive interstitial lung disease: a systematic review and meta-analysis. Ann Med. 2024;56(1):2411605. 39382564 10.1080/07853890.2024.2411605 PMC11465402

[105] Li J, Wang S, Zheng J, Li Q, Li J, Lu L. Clinical characteristics of and risk factors for Pneumocystis jirovecii pneumonia in anti-melanoma differentiation-associated gene 5 (Anti-MDA5) antibody-positive dermatomyositis patients: a single-center retrospective study. Clin Rheumatol. 2023;42(2):453–62. 36301369 10.1007/s10067-022-06403-9

[106] Chen X, Shu X, He L, Yang H, Lu X, Wang G, et al. High prevalence and mortality of Pneumocystis jirovecii pneumonia in anti-MDA5 antibody-positive dermatomyositis. Rheumatology (Oxford). 2023;62(10):3302–9. 36734589 10.1093/rheumatology/kead063

[107] Liu L, Zhang Y, Liu S, Wang C, Zhang L, Guan W, et al. Compounded sulfamethoxazole improved the prognosis of dermatomyositis patients positive with anti-melanoma differentiation-associated gene 5. Rheumatology (Oxford). 2023;62(9):3095–100. 36702462 10.1093/rheumatology/kead034

[108] Hayashi M, Aoki A, Asakawa K, Sakagami T, Kikuchi T, Takada T. Cytokine profiles of amyopathic dermatomyositis with interstitial lung diseases treated with mycophenolate. Respirol Case Rep. 2017;5(4):e00235. 28413686 10.1002/rcr2.235 PMC5387408

[109] Romero-Bueno F, Diaz Del Campo P, Trallero-Araguás E, Ruiz-Rodríguez JC, Castellvi I, Rodriguez-Nieto MJ, et al. Recommendations for the treatment of anti-melanoma differentiation-associated gene 5-positive dermatomyositis-associated rapidly progressive interstitial lung disease. Semin Arthritis Rheum. 2020;50(4):776–90. 32534273 10.1016/j.semarthrit.2020.03.007 PMC11616672

[110] Zhang X, Zhou S, Wu C, Li M, Wang Q, Zhao Y, et al. Tocilizumab for refractory rapidly progressive interstitial lung disease related to anti-MDA5-positive dermatomyositis. Rheumatology (Oxford). 2021;60(7):e227–8. 33410494 10.1093/rheumatology/keaa906

[111] Qiu L, Shao X, Ma L, Fan Z, Yu H. Successful Tocilizumab treatment for rapidly progressive interstitial lung disease with anti-MDA5-positive juvenile dermatomyositis: a case report and literature review. Front Pediatr. 2024;12:1497168. 39664278 10.3389/fped.2024.1497168 PMC11631605

[112] Zheng Q, Wang Z, Tan Y, Zhu K, Lu M. Over activation of IL-6/STAT3 signaling pathway in juvenile dermatomyositis. Rheumatol Ther. 2024. 10.1007/s40744-024-00699-6 PMC11422336 39073510

[113] Niu Y, Liu S, Qiu Q, Fu D, Xiao Y, Liang L, et al. Increased serum level of IL-6 predicts poor prognosis in anti-MDA5-positive dermatomyositis with rapidly progressive interstitial lung disease. Arthritis Res Ther. 2024;26(1):184. 39468670 10.1186/s13075-024-03415-5 PMC11520069

[114] Habib T, Senadheera S, Weinberg K, Kaushansky K. The common gamma chain (gamma c) is a required signaling component of the IL-21 receptor and supports IL-21-induced cell proliferation via JAK3. Biochemistry. 2002;41(27):8725–31. 12093291 10.1021/bi0202023

[115] Asao H, Okuyama C, Kumaki S, Ishii N, Tsuchiya S, Foster D, et al. Cutting edge: the common gamma-chain is an indispensable subunit of the IL-21 receptor complex. J Immunol. 2001;167(1):1–5. 11418623 10.4049/jimmunol.167.1.1

[116] Wang Y, Zou R, Wei J, Tang C, Wang J, Lin M. The efficacy and safety of Tofacitinib in anti-melanoma differentiation-associated gene 5 antibody positive dermatomyositis associated interstitial lung disease: a systematic review and meta-analysis. Ther Adv Respir Dis. 2024;18:17534666241294000. 39480695 10.1177/17534666241294000 PMC11528585

[117] Chen Z, Wang X, Ye S. Tofacitinib in amyopathic Dermatomyositis-Associated interstitial lung disease. N Engl J Med. 2019;381(3):291–3. 31314977 10.1056/NEJMc1900045

[118] Ida T, Furuta S, Takayama A, Tamura J, Hayashi Y, Abe K, et al. Efficacy and safety of dose escalation of Tofacitinib in refractory anti-MDA5 antibody-positive dermatomyositis. RMD Open. 2023;9(1):e002795. 36593080 10.1136/rmdopen-2022-002795 PMC9809321

[119] Huang X, Zhang G, Luo S. A case of refractory anti-MDA5-positive amyopathic dermatomyositis successfully treated with Upadacitinib. J Dermatolog Treat. 2024;35(1):2391445. 39191432 10.1080/09546634.2024.2391445

[120] Harada H, Shoda H, Tsuchiya H, Misaki M, Sawada T, Fujio K. Baricitinib for anti-melanoma differentiation-associated protein 5 antibody-positive dermatomyositis-associated interstitial lung disease: a case series and literature review on Janus kinase inhibitors for the disease. Rheumatol Int. 2024;44(5):961–71. 38456909 10.1007/s00296-024-05551-2 PMC10980644

[121] Ge Y, Li S, Tian X, He L, Lu X, Wang G. Anti-melanoma differentiation-associated gene 5 (MDA5) antibody-positive dermatomyositis responds to rituximab therapy. Clin Rheumatol. 2021;40(6):2311–7. 33411136 10.1007/s10067-020-05530-5

[122] Clottu A, Laffitte E, Prins C, Chizzolini C. Response of mucocutaneous lesions to rituximab in a case of melanoma differentiation antigen 5-related dermatomyositis. Dermatology. 2012;225(4):376–80. 23428928 10.1159/000346573

[123] Koichi Y, Aya Y, Megumi U, Shunichi K, Masafumi S, Hiroaki M, et al. A case of anti-MDA5-positive rapidly progressive interstitial lung disease in a patient with clinically amyopathic dermatomyositis ameliorated by rituximab, in addition to standard immunosuppressive treatment. Mod Rheumatol. 2017;27(3):536–40. 25698373 10.3109/14397595.2015.1014140

[124] Mao MM, Xia S, Guo BP, Qian WP, Zheng ZX, Peng XM, et al. Ultra-low dose rituximab as add-on therapy in anti-MDA5-positive patients with polymyositis /dermatomyositis associated ILD. Respir Med. 2020;172:105983. 33032789 10.1016/j.rmed.2020.105983

[125] Holzer MT, Nies JF, Oqueka T, Huber TB, Kötter I, Krusche M. Successful rescue therapy with daratumumab in rapidly progressive interstitial lung disease caused by MDA5-Positive dermatomyositis. Chest. 2023;163(1):e1–5. 36628678 10.1016/j.chest.2022.08.2209

[126] Ostendorf L, Muench F, Thormählen L, Galbavý Z, Körner R, Nee J, et al. Rescue combination treatment of anti-MDA5-associated ARDS with daratumumab. RMD Open. 2023;9(3):e003238. 37479497 10.1136/rmdopen-2023-003238 PMC10364176

[127] Chua CG, Chai GT, Lim XR, Manghani M, Leung BPL, Koh LW. Successful rescue treatment of refractory anti-MDA5 autoantibody positive dermatomyositis with rapidly progressive interstitial lung disease using daratumumab. Clin Exp Rheumatol. 2024;42(2):460–1. 38293965 10.55563/clinexprheumatol/monpb6

[128] Guo Y, Liu H, Chen B, Zhang K, Meng L, Yan L, et al. Dysregulated CD38 expression on T cells was associated with rapidly progressive interstitial lung disease in anti-melanoma differentiation-associated gene 5 positive dermatomyositis. Front Immunol. 2024;15:1455944. 39588376 10.3389/fimmu.2024.1455944 PMC11586385

[129] Zou J, Li T, Huang X, Chen S, Guo Q, Bao C. Basiliximab May improve the survival rate of rapidly progressive interstitial pneumonia in patients with clinically amyopathic dermatomyositis with anti-MDA5 antibody. Ann Rheum Dis. 2014;73(8):1591–3. 24739327 10.1136/annrheumdis-2014-205278

[130] Hagen M, Bucci L, Böltz S, Nöthling DM, Rothe T, Anoshkin K, et al. BCMA-Targeted T-Cell-Engager therapy for autoimmune disease. N Engl J Med. 2024;391(9):867–9. 39231353 10.1056/NEJMc2408786

[131] Bando T, Yamano Y, Takei R, Sasano H, Fukihara J, Yokoyama T et al. Early intervention of antifibrotics along with anti-inflammatory treatment in rapidly progressive interstitial lung disease (RP-ILD) with anti-MDA5 antibody-positive dermatomyositis (MDA5-DM). European Respiratory Journal [Internet]. 2023 Sep 9 [cited 2024 Jan 30];62(suppl 67). Available from: https://erj.ersjournals.com/content/62/suppl_67/PA401

[132] Puthumana RM, Koch AL, Schettino C, Vehar SJ. Asymptomatic and slowly progressive anti-MDA5 ILD: A report of three cases deviating from a notoriously rapidly progressive ILD. Respir Med Case Rep. 2024;51:102072. 39040087 10.1016/j.rmcr.2024.102072 PMC11261436

[133] Lian QY, Chen A, Zhang JH, Xu X, Huang DX, Luo Q, et al. Lung transplantation for anti-MDA5-positive dermatomyositis-associated rapid progressive interstitial lung disease: report of two cases and review of the literature. Clin Rheumatol. 2023;42(3):941–7. 36441397 10.1007/s10067-022-06422-6

[134] Allenbach Y, Uzunhan Y, Toquet S, Leroux G, Gallay L, Marquet A, et al. Different phenotypes in dermatomyositis associated with anti-MDA5 antibody: study of 121 cases. Neurology. 2020;95(1):e70–8. 32487712 10.1212/WNL.0000000000009727 PMC7371381

[135] Xu L, You H, Wang L, Lv C, Yuan F, Li J, et al. Identification of three different phenotypes in Anti-Melanoma differentiation-Associated gene 5 Antibody-Positive dermatomyositis patients: implications for prediction of rapidly progressive interstitial lung disease. Arthritis Rheumatol. 2023;75(4):609–19. 35849805 10.1002/art.42308

[136] Guo R, Yang Y, Gu L, Li X, Ma Y, Liu X, et al. Disease-associated immune cell endotypes in anti-MDA5-positive dermatomyositis using unbiased hierarchical clustering. Front Immunol. 2024;15:1349611. 38533498 10.3389/fimmu.2024.1349611 PMC10963492

