# Supplementary material for: Anti- Melanoma Differentiation-Associated Gene 5 Antibody Positive Dermatomyositis: Recent Progress in Pathophysiology and Treatment
Source: Curr Rheumatol Rep. 2025 May 5;27(1):23. doi: 10.1007/s11926-025-01188-7 (PMC12052924; doi:10.1007/s11926-025-01188-7)
Supplement: Supplementary file 1 — Supplementary Material 1 [file 11926_2025_1188_MOESM1_ESM.docx]

**Anti-** **melanoma differentiation-associated gene 5 Antibody Positive Dermatomyositis: Recent Progress in Pathophysiology and Treatment**

Tsuneyasu Yoshida1,2* MD, Ran Nakashima1 MD, PhD.

1 Department of Rheumatology and Clinical Immunology, Graduate School of Medicine,

Kyoto University, Kyoto, Japan.

2 Division of Clinical Immunology and Cancer Immunotherapy, Center for Cancer Immunotherapy and Immunobiology, Graduate School of Medicine, Kyoto University, Kyoto, Japan.

**ABSTRACT**

**レビューの目的:**抗黒色腫分化関連遺伝子 5 (MDA5) 抗体陽性皮膚筋炎 (MDA5-DM) は、臨床的に無筋症状を呈しやすく、急速に進行する間質性肺疾患との高リスク関連を特徴とする、全身性自己免疫疾患である。しばしば免疫抑制治療に抵抗性を示し致命的となるが、その病態メカニズムは完全には解明されていない。このレビューでは、MDA5-DMに関する最近の研究の進歩を包括的にまとめ、疾患メカニズムへの理解を深めることで、新たな治療戦略の開発に寄与する次の基礎研究を加速させることである。

**最近の知見：**最近の進歩により、疾患感受性に寄与する遺伝的要因と環境的要因、免疫病理学的プロセスやサイトカインネットワークなど、この疾患のさまざまな側面が明らかになってきた。さらに、長い間議論されてきた抗MDA5抗体の病原性や認識エピトープ、産生機序に関する理解も進んだ。治療の面では、古典的な3剤併用療法に加えて、JAK阻害剤とリツキシマブの新たな有効性が認められている。リンパ球を標的とする生物学的製剤の開発により、治療オプションの進歩へのさらなる希望が生まれている。

**概要:** MDA5-DM の最新の病態生理学の理解が進むことにより、より安全で効果的な治療戦略が開発される事を望む。

**INTRODUCTION**

抗抗黒色腫分化関連遺伝子5 (MDA5) 抗体陽性皮膚筋炎（MDA5-DM）は、皮膚、肺、血管に炎症を引き起こす全身性自己免疫疾患である。皮膚筋炎における割合は、Caucasianでは全体の2%未満だが、Asianではその10倍を超える20％以上を占める(1–3)。さらに有病率もCaucasianよりAsianで高い事が特徴的である(4)。

MDA5-DMの特徴は、筋肉の障害の徴候が乏しい筋無症候性皮膚筋炎（CADM）の病型を示す頻度が高いことである。また、高率に治療抵抗性の急速進行性の間質性肺炎（RP-ILD）を合併し、6か月間死亡率が28-66%と、最も予後不良な自己免疫疾患である(5–7)。しかし、この致死的なRP-ILDの頻度も人種差があり、AsianではMDA5-DMのうち、50-100%がRP-ILDを合併するが、Caucasianでは20-57％とより低い頻度で合併する(7–12)。

MDA5-DMに伴う致死的なRP-ILDに対して新規治療法の開発が望まれるが、その病態や病因機序の把握は未だに十分ではない。しかし、近年、基礎研究を中心に、MDA5-DMに関わる報告が続いており、我々は確実に疾患の辺縁からMDA5-DMの謎を紐解きつつある。

本レビューでは、MDA5-DMの最新の知見を包括的する事で、病態の理解や新規治療法探索に関わる基礎研究の発展に貢献する事を目標とする。

1. **MDA5-DMの要因**

近年の目覚ましい研究により、他のリウマチ膠原病疾患と同様に、MDA5-DMの発症には遺伝的要因と環境要因が複合的に関与していることが示唆されている。遺伝的要因に関しては、MDA5-DMの疾患感受性に関与する遺伝子変異の存在や、その変異の発現が人種間で異なることも同定されている。環境要因に関しては、特定のウイルスがMDA5-DMの発症に関連し、そのトリガーとして注目されるようになっている。

- 1. **遺伝的要因**

MDA5-DMの遺伝的要因として、従来の日本からの報告では、human leukocyte antigen (HLA)の一部であるHLA-DRB1*0101および*0405が疾患感受性と関連していることが示されている(13)。HLA-DRはMHCクラスIIに相当し、抗原提示細胞が抗原ペプチドをT細胞に提示する役割を果たす。これらのHLA分子が自己免疫疾患において重要となる理由は、自己抗原を免疫系に提示することで自己抗体の産生を誘導する機序に関与しているためである。

例えば、関節リウマチでは、特定のHLAハプロタイプやアレルがシトルリン化ペプチドと強い結合能を持ち、その結合がT細胞への抗原提示や抗CCP抗体の産生に関連していることが知られている。MDA5-DMでも同様に、特定のHLA-DRB1*0101および*040アレルがMDA5ペプチドをT細胞に提示することで、抗MDA5抗体の産生感受性に関与している可能性が示唆される。興味深いことに、Caucasianを対象とした研究では、MDA5-DMに有意に関連するHLAが同定されていない(14)。後述するように、人種間で抗MDA5抗体が認識するMDA5のエピトープ部位が異なることが知られているが、人種間のHLAハプロタイプの違いが、MDA5抗体の陽性頻度や抗原エピトープのレパートリーの違いを生じさせている可能性があり、さらには、MDA5-DMの人種的な罹患頻度や表現型の違いを引き起こしている可能性が考えられる。

非HLA関連遺伝子変異として、ゲノムワイド関連解析から、WDFY4遺伝子の短縮型変異体（truncated variant, rs7919656）が、CADMおよびRP-ILDとの関連を持つことが明らかになっている(15,16)。古典的樹状細胞は本来CD4^+^T細胞へ抗原提示を行うが、WDFY4の細胞内での役割により、抗原をCD8^+^T細胞へクロスプレゼンテーションする事が可能となり(17)、CD8^+^T細胞も活性化させている可能性がある。また、WDFY4はMDA5を介したNF-κBシグナル伝達を増幅すること(15)、p53を介してCD8^+^T細胞のアポトーシスを制御する事が報告されている(18)。WDFY4の変異や機能異常が、自己免疫応答に寄与する可能性が示唆される。

WDFY4変異体はヨーロッパ人では確認されていないものの、日本人や中国人などアジア人のMDA5-DM患者に見られ、RP-ILDとの関連が報告されている(15,16)。HLAと合わせて、遺伝的変異の人種差が、CaucasianとAsianにおけるMDA5-DMの罹患頻度や重症度の違いに関わっている可能性が示唆される(19)。

- 1. **環境要因**

MDA5-DMの発症が郊外や河川の近くに住む住民で多い傾向は既に指摘されて来た(6)。近年では、冬から春にかけてMDA5-DMの発症が多い事が人種に寄らず報告されている(6,20–23)。MDA5-DMの発症と地理、季節との関連は、環境に由来する何らかの因子が疾患発症に関与している可能性を示唆する。特に、MDA5が様々なウイルスを認識する細胞内のウイルスセンサーである特性を踏まえると、ウイルス感染がMDA5-DM発症に関わる重要な環境要因である可能性が考えられる。

MDA5はウイルスを感知すると、抗ウイルス作用を持つtype I IFN産生を促すが、興味深い事に、type I IFN自体がMDA5発現の上方に調節する事が報告されている(24–26)。すなわち、何らかのウイルス感染をトリガーとしてMDA5が活性化し、type I IFNの産生が亢進する事で、さらにMDA5発現が亢進する可能性がある。MDA5-DM患者では、このポジティブフィードバックループが肺や皮膚などの局所の炎症部位で形成される事で、特徴的な臓器障害の形成に寄与していると考えられる。

MDA5-DM患者の循環単球を用いたintegrated miRNA-mRNA association解析では、type I IFN炎症反応の上流調節因子としてTLR3、TLR7、TLR9といったウイルス遺伝子を認識する受容体を介したシグナルがPU.1による転写調節と相まって、type I IFN媒介炎症反応とCCL2の上方制御分泌を促進し、最終的に抗ウイルス炎症反応につながる事が同定された(27)。この事は、TLR3/7/9のリガンドとなる一本鎖および二本鎖RNAウイルス感染が上流調節因子を活性化する事で、MDA5-DMの病態を誘発する可能性を示唆する。

元来、ピコルナウイルスを認識する役割を持つMDA5だが、MDA5-DMの発症と特定のウイルス感染との明確な関連を示す報告は限られていた。しかし、Gene Set Enrichment解析の結果から、単純ヘルペスウイルス1型（HSV-1）感染経路に関連する遺伝子発現が皮膚筋炎間質性肺炎患者で亢進していることが示され、さらにMDA5-DM患者ではHSV-1の蛋白質合成を阻害する遺伝子群の発現が顕著に増加していることが報告されている(28)。これらの結果は、MDA5-DM患者においてHSV-1感染への免疫応答が活性化していることを示唆している。さらにMDA5-DM患者血清を用いた別の報告では、エンテロウイルス B ペプチドに対する抗体反応が増加している事を見出した(29)。

これらの知見を統合すると、MDA5-DMの病態においてHSV-1やエンテロウイルス Bなどの何らかのウイルス感染が、MDA5ならびにTLR刺激を介してtype I IFN経路を活性化させるトリガーとして働く可能性を示唆するものである(Figure 1)。

**Figure 1: Genetic and environmental factors of MDA5-DM**


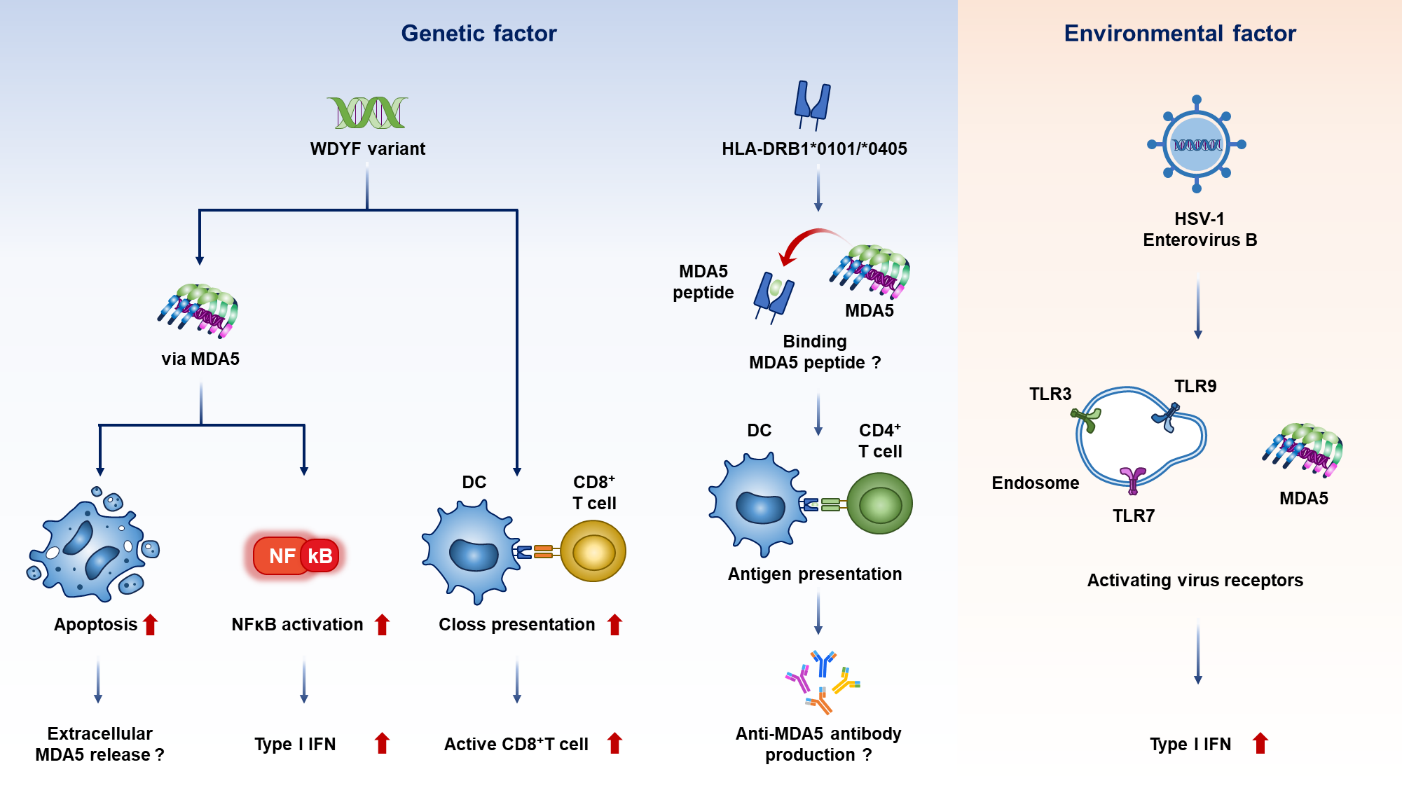


1. **MDA5-DMの免疫病態**
   1. **Type I interferonopathy and macrophage activation**

MDA5-DMの病態に最も関わるのはtype I IFNである。患者血清でtype I interferonopathyが観察されるだけでなく(30,31)、トランスクリプトーム解析の結果でも、患者の肺や皮膚、血管組織においてtype I IFNシグナルが顕著に活性化していることが示されている(32,33)。Type I IFNの活性化は頻発する血管障害とも関連が指摘されており(34)、MDA5-DMにおける重要な炎症トリガーとしての役割を担う。さらに疾患の予後不良とも密接に関連している(35)。

Type I IFNの主要な供給源としては、マクロファージや樹状細胞が注目されている(36)。特にマクロファージの活性化は、患者血清のプロテオミクス解析の結果で明らかとなっている。フェリチンやキトトリオシダーゼ、sCD163、Galectin-9などのマクロファージ由来または貯蔵分子が患者血清中で高値を示し、予後予測因子としての有用性を持つことに加えて(37–40)、その活性化因子サイトカインやケモカインであるCXCL10 / IP-10やIL-34がMDA5-DMで高値を示している(41,42)。病理学的には、活性化したマクロファージは傷害を受けた臓器内で確認されているが(43)、末梢血と肺胞洗浄液(BALF)中の免疫細胞を比較したシングルセルRNA解析の結果では、この肺内で活性化したマクロファージが主にtype I IFNを含めた炎症性サイトカインを産生している事が示されている(44)。

- 1. **Type II and type III interferon**

Type I IFNに加えて、type II IFN（IFN-γ）およびtype III IFN（IFN-λ）も血清中で上昇することが報告されている(33,45–47)。

DM患者におけるRP-ILDの形成には、T細胞やNK細胞から供給されるIFN-γが関与するとの報告がある(48)。さらに、MDA5-DM患者の自己抗体が直接的に末梢血細胞におけるIFN-γの産生を促進することも明らかにされている(49)。加えて、IFN-γはIL-1βと組み合わさることで、ヒト肺線維芽細胞によって分泌され、マクロファージ遊走性ケモカインである可溶性CX3CL1(フラクタルカイン)を誘導することが報告されている(50,51)。皮膚ではケラチノサイトからIFNγ誘導性にIL-18が分泌され、皮膚病変の病態形成にも関わっている事が報告されている(52)。

一方で、上皮バリア表面において抗ウイルス機能を持つtype III IFN（IFN-λ）のうち、IFN-λ3の高値は、MDA5-DM患者の皮疹やILDの活動性および予後不良を予測する因子であるとされている(47)。IFN-λはまた、細胞外からJAK/STAT1経路を介して、様々な自己免疫疾患で病原性を有するdouble negative 2 B細胞の分化を誘導し、MDA5-DMの病態形成に寄与する可能性が指摘されている(53)。

- 1. **T cell**

MDA5-DM患者の末梢血では、CD4^+^T細胞やCD8^+^T細胞、CD4^+^/CD8^+^比が重症例で減少し(54–56)、さらにT細胞の経時的な減少は、死亡率の増加とも関連する事が報告されている(57)。末梢血中のリンパ球減少は、骨髄でのリンパ球分化の異常は見られていない事から(54)、分化成熟後の減少、あるいは障害臓器への集簇が疑われている。

重症患者の肺胞洗浄液中の免疫細胞を検討した報告では、ISG15^+^CD4^+^T細胞やISG15^+^CD8^+^T細胞などのIFNシグネチャーの活性化したT細胞が組織に浸潤していることが示されている(33,54)。さらに末梢血とBALF中のT細胞受容体配列決定を解析した結果では、肺内のT細胞は末梢と比較してより著明なクローン増殖を示し、末梢血T細胞と対となるクロノタイプを共有していた(44)。これは、T細胞が末梢血から肺へリクルートされている可能性を示唆している。

一方、MDA5-DMを含めた皮膚筋炎においてRIG-I pathwayの過剰発現がT細胞のアポトーシスを誘導し、in vitro での T細胞の増殖を阻害する事も知られている(58)。また、末梢血T細胞においてアポトーシス、ピロトーシス、ネクローシスなどの細胞死経路の遺伝子が顕著に増加している事も報告されており(44)、重症患者においてT細胞が細胞死を起こして減少している可能性が示唆される。

- 1. **B cell**

MDA5-DM患者血清ではB細胞の活性化が見られる。患者血清ではBAFFが高値を示し(59,60)、末梢血ではCD19^+^B細胞が増加し活性化している(33,61)。B細胞のサブセットでは、メモリーB細胞が減少し、形質芽細胞が増加して活性化している事が判明している(62)。他の自己免疫疾患と同様に、MDA5-DMでも抗Ro52抗体が共存するとRP-ILD の頻度が増加し、表現型がより予後不良となる報告がある(63–65)。さらに一部の患者では自然免疫応答に関与することが知られているスプライシング因子プロリン/グルタミンリッチタンパク質(SFPQ)に対する抗体が出現している(66)。これらの自己抗体の出現は、B細胞系統の寛容破綻が病態悪化に関わっている可能性を示唆する。なお、Ro52に関してはE3ユビキチンリガーゼ活性を持ち、インターフェロン調節因子（IRF）3とIRF7のユビキチン化に関与しており(67)、type I IFN産生を抑制する役割を有する(68,69)。In vitro実験では、抗Ro52抗体がRo52の生物活性を阻害することによってtype I IFNシグナル伝達を増幅するとされるが、さらなる検討が必要である(70)。

- 1. **Mitochondria**

MDA5は細胞内でミトコンドリア表面上のレセプターを介してtype I IFNを産生するため、MDA5-DMにおけるミトコンドリア異常が調査されている。MDA5-DM患者の筋生検検体を用いた検討では、高いミトコンドリア異常率を認め、MDA5、MAVS、IRF7、ISG15の発現レベルが対照群よりも高かったという(71)。しかしながら、MDA5-DMの筋病変の頻度は他の皮膚筋炎と異なり、決して高くない。さらに最も罹患頻度の高い肺病変や皮膚病変ではミトコンドリア異常は同定されておらず、今後の追加の検討が必要である。

- 1. **Fibrosis**

MDA5-DM の肺病変では、線維化が生じるかについて注目が集まっている。MDA5-DMの血清サイトカイン解析によると、炎症性サイトカイン(IL-1、IL-6、TNF-α、IL-18)に加えて、線維化に関わるサイトカインであるIL-8(CXCL8)やIL-10が亢進している(37,45)。また、肺線維芽細胞の増殖、移動、コラーゲン産生を促進することで肺線維症を促進するSoluble CXCL16値が有意に高く、RP-ILDの予後予測因子となる(44)。MDA5-DM患者の肺組織シングルセル解析の結果では、線維化に関連する遺伝子発現が亢進している事が示されており(33)、剖検症例報告でも、RP-ILDの肺組織にはCD68やCD163が陽性で、フェリチンを高産生する活性化したM2型肺胞マクロファージが肺に集簇しており、病態に関わるようである(39,43,73)。さらに、患者の末梢血や肺胞洗浄液の解析では、肺において肺胞マクロファージや気道上皮細胞がstromal cell derived factor-1 (SDF-1)を産生して、IL-21を高発現し、肺線維化を促進させるCD4^+^CXCR4^+^T細胞を呼び寄せると言われている(74)。CD4^+^CXCR4^+^T細胞は、肺線維芽細胞の増殖とTGF-β、α-SMA、およびコラーゲンIの産生を促進させるが、肺線維芽細胞自身もまたIFNγやIL-1βの影響受けて前述のようにCX3CL1を発現し、CX3CR1^+^ M2マクロファージを集簇させ、肺線維症を促進させる可能性がある(51)。既報では、IL-21はさらにIL-13を産生する線維化促進性CD8^+^T細胞を介して肺線維症を促進させることが報告されている(75)。これは肺組織のシングルセル解析で認めた、肺線維症に関わるISG15^+^CD8^+^T細胞などの特徴的なT細胞の増加の結果と一致する(33)。また、間葉系細胞から分泌される抗線維化作用のあるエクソソームmicroRNA-30bの発現がMDA5-DMの生検肺組織では低下している報告もある(76)。これらの結果を統合すると、MDA5-DMの肺病変では線維化が進行する分子学的なポテンシャルが有しているようである。

しかしながら、臨床的にはMDA5-DMの肺病変では抗ARS抗体症候群や特発性肺線維症ほどの線維化が見られない事に留意しなければならない。放射線学的にMDA5-DMの肺病変は器質化肺炎が主であり(73)、急性期の場合、HRCTにおいて線維化所見が乏しい事が知られている(77)。血清のサイトカイン解析では線維化抑制に関わるCXCL10(IP-10)が上昇している報告もある(37,45)。興味深い事に、in vitroの実験ではヒト胎児肺線維芽細胞にMDA5を過剰に発現させた場合、TGF-β signaling pathwayを介した線維化が抑制される事が示されている(77)。

このように、潜在的な分子学的線維化のポテンシャルが、何故臨床像に顕著に現れて来ないのか、炎症と修復のバランスの観点から、更なる研究が必要である。

1. **Advances of research in anti-MDA5 antibody**

　近年、抗MDA5抗体価の病原性への関与の可能性について関心が高まっている。多くのin vitro研究により、抗MDA抗体の病原性が示されるようになった(Figure 2)。さらに抗MDA5抗体のサブクラスが判明し、さらに認識する部位に人種差があり、臨床像と関わる事も知られて来た。

**Figure 2. Presumed immunopathogenesis of anti-MDA5 antibodies**


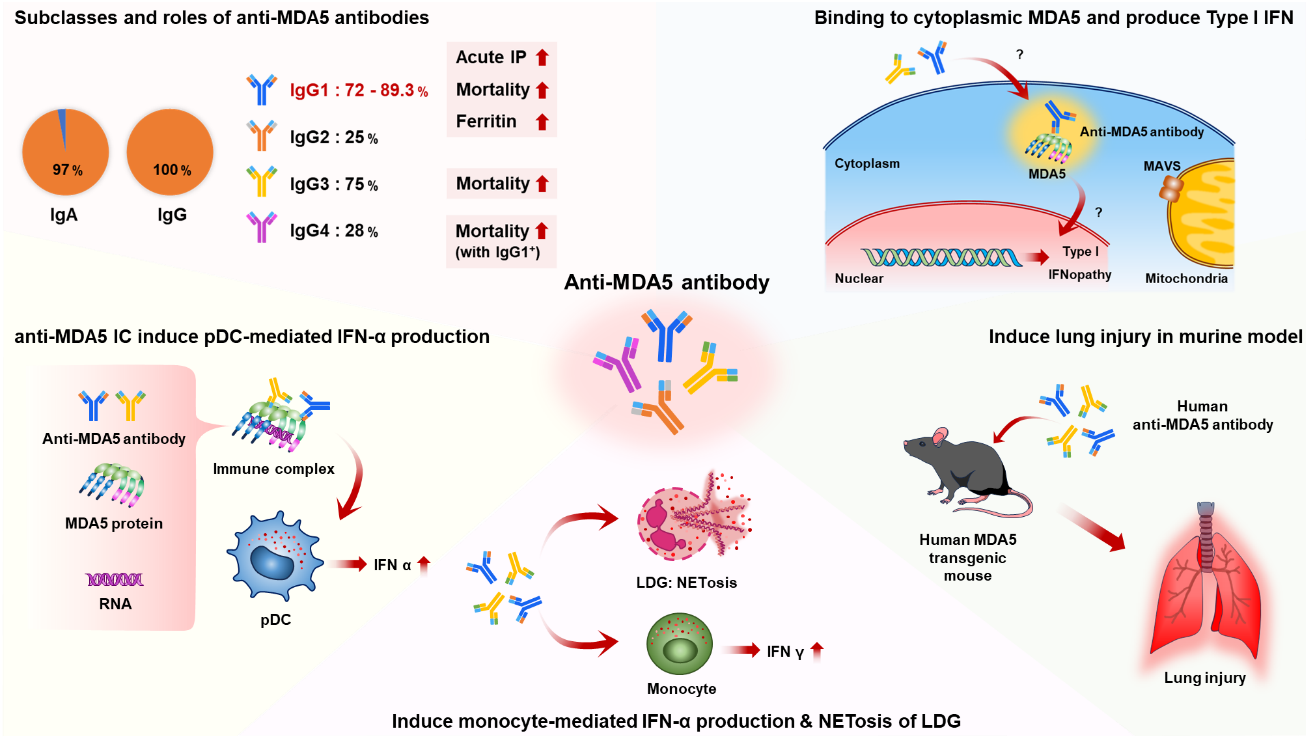


- 1. **抗MDA5抗体の病原性**

抗MDA5抗体価は、type I IFNシグネチャーと強い相関関係を示し(78)、MDA5-DM の疾患活動性と関連することが明らかとなっている(79)。さらに、RP-ILDを伴うCADM患者における予後予測因子として位置づけられている(7,19)。加えて、MDA5-DMの生存患者では寛解後に抗MDA5抗体価が低下する一方(80)、非生存患者では低下しない事が報告されている(81)。また、重症患者に対して血漿交換療法が有効であり、血漿交換療法後に生存した患者では抗MDA5抗体価が低下した事(37)から、 抗MDA5抗体の病原性が疑われ、除去することで病態が改善する可能性が示唆されている。

In vitro実験において、抗MDA5抗体を好中球に添加するとneutrophil extracellular trapsを引き起こす事が知られている(82)。MDA5-DM患者の B 細胞クローンによって産生された抗MDA5モノクローナル抗体をさらにPBMCの単球に添加するとIFNγ産生が亢進する事も判明した(49)。抗MDA5抗体を含む免疫複合体の病原性も指摘されており、人工的に生成した抗MDA5抗体、MDA5蛋白、RNAを含む免疫複合体を形質細胞様樹状細胞に添加すると、TLR-7を介してIFN-αを強力に誘導する事が実験レベルで判明した(83)。Electroporateされた筋細胞に患者から精製した抗MDA5抗体を添加した実験では、抗MDA5抗体が細胞質のMDA5に結合し、type I IFNであるIFNβ1およびその誘導遺伝子の発現が過剰となることが確認された(84)。この実験において肺細胞や血管内細胞については未検討で、抗MDA5抗体がどのように細胞質に侵入するのかについては追加の検討が必要であるが、細胞質のMDA5に抗MDA5抗体が結合する事も、type I interferonopathyを引き起こす原因の一つである可能性を示唆する重要な知見であるといえる。

動物モデルでは、ヒトのMDA5蛋白質を過剰発現したマウスに、ヒト抗MDA5抗体を投与した研究では、重度の肺炎症細胞浸潤が観察された(85)。興味深い事に、野生型マウスに抗ヒトMDA5ポリクローナル抗体を投与しても肺炎が生じなかったことである。一方、野生型マウスでも、マウス組み換え全長MDA5蛋白質を免疫する事で抗MDA5抗体を産生させた上で、MDA5を刺激するウイルスに類似した人工二本鎖RNA（ポリイノシン-ポリシチジル酸）を経鼻投与した所、急性肺障害が誘発された(86)。これらの結果は、抗MDA5抗体の存在はそれ自体が肺炎を惹起するものではないが、抗体の存在下で何らかのウイルス感染によって肺局所でMDA5が過剰に発現・活性化した場合、肺炎症が増悪することを示唆している。

- 1. **抗MDA5抗体のサブクラス**

抗MDA5抗体のサブタイプとサブクラスに関する研究も進んでいる。検出される抗MDA5抗体のサブタイプの大多数はIgG型(100%)であるが、興味深い事に、IgA型抗MDA5抗体も97%の患者で同時に検出される(87)。しかしながら、その臨床的な意義はまだ不明である。

IgGのサブクラスについての研究では、検出頻度に差異が見られるものの、共通してIgG1型が最も高頻度で検出され、予後不良と関連する事が確認されている(87,88)。IgG1サブクラスはIgGの中でも特に炎症を誘発する事で知られており、炎症誘発性サイトカイン(TNF、IL-1β、IL-23など)の産生を促進し、type I IFN応答を活性化することが知られている(89)。さらに、IgG1は一般的に補体も活性化させ、補体を介した細胞傷害や免疫複合体形成の可能性も示唆されている。

IgG1に次いで高頻度で検出されるIgG3型、そしてIgG1型と共にIgG4型も陽性の場合、予後不良と関連が強まる事が示唆されている(87,88)。これらの知見は、抗MDA5抗体のサブタイプ、特にIgG型抗MDA5抗体のサブクラスが疾患の予後に重要な影響を与える可能性があることを示しているが、さらなる検証が必要である。

- 1. **抗MDA5抗体の認識部位**

近年、抗MDA5抗体が認識するMDA5のエピトープに関する研究が注目されている。中国人やEuropean患者の血清に含まれる抗MDA5抗体と、細分化されたMDA5蛋白質およびペプチドとの結合能を調べた研究では、MDA5の3つのドメインのうち、ヘリカーゼドメインが抗MDA5抗体の主要なエピトープであることが明らかとなった(90,91)。一方で、別の報告によれば、日本人ではCARDドメインのフラグメントを認識する抗MDA5抗体が多く検出されるのに対し、AmericanではCTDドメインのフラグメントを認識する抗体が多く見られるという(92)。

さらに、抗MDA5抗体が認識するエピトープ部位の違いによって、臨床像が異なることも報告されている。例えば、女性では男性に比べてCTDドメインを認識する抗MDA5抗体が多く検出される傾向があり、筋病変や血管病変を有する患者ではヘリカーゼドメインを認識する抗MDA5抗体が多いとされる(93)。また、間質性肺炎の進行によって死亡した患者では、CARDドメインのフラグメントを認識する抗MDA5抗体が多く検出された。このように、抗MDA5抗体の認識するエピトープの違いが人種間の致死率や臨床フェノタイプの差異に寄与している可能性が示唆されている(Figure 3)。


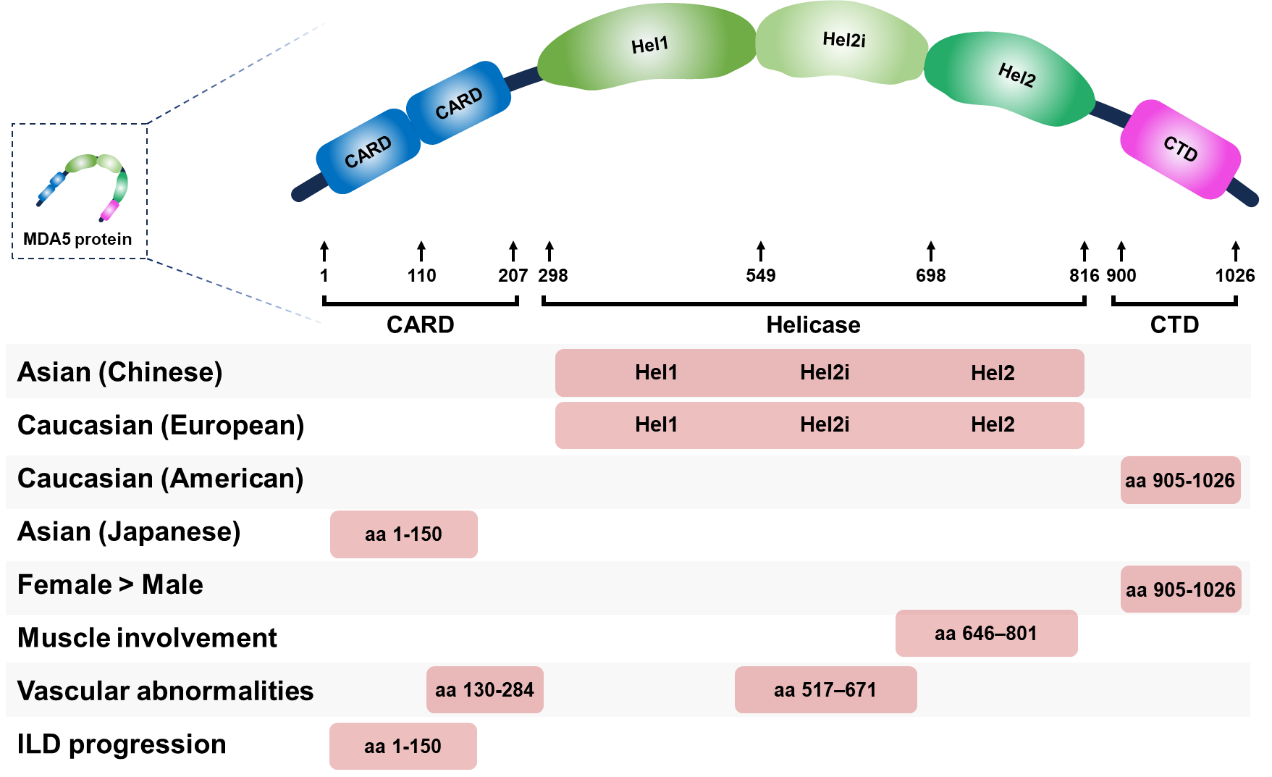
**Figure 3. Association between clinical phenotypes and recognition epitopes of anti-MDA5 antibodies**

- 1. **抗MDA5抗体の産生機序**

抗MDA5抗体の病原性や認識エピトープに関する理解が進む一方で、産生機序についても注目が高まっている。MDA5-DM患者において再燃時に肺癌が疑われた結節を切除した後、抗MDA5抗体が自然と正常化した症例報告がある(94)。この事例は、抗MDA5抗体の産生源が肺である可能性を示唆するものである。また、少数のケースレポートによれば、抗MDA5抗体の陽転化はMDA5-DMの発症前から始まっている可能性が示唆されている(81)。同種造血細胞移植後に発症したMDA5-DM患者4名の血清を遡及的に分析した研究では、1名を除き抗MDA5抗体がILD発症の43～99日前に陽性化していた(95)。これは、関節リウマチにおける抗CCP抗体と同様に、抗MDA5抗体が発症前から陽転化しているpre-clinicalなステージが存在する可能性を示唆している。

抗MDA5抗体の産生が進むためには、通常細胞質内に存在しているMDA5が何らかの機序で細胞外に放出され、抗原提示細胞に認識される必要がある。前述の通り、MDA5-DM患者ではtype I IFNによって肺や皮膚の上皮細胞、末梢血中の好中球や単球においてMDA5の発現が亢進している(24–26)。このような間葉系細胞や免疫細胞が細胞死を起こすことで細胞質内のMDA5が放出され、抗原提示細胞に認識される可能性が考えられる。

　一方で、興味深いことに、皮膚筋炎患者の末梢血において末梢血単核球から恒常的にMDA5蛋白質が分泌されていることが示されている(96)。さらに、末梢血単核球にMDA5が認識する二本鎖RNAを添加すると、この分泌が促進されることが明らかになった。このことから、例えばウイルス感染がきっかけとなり、末梢血単核球におけるMDA5発現が亢進するだけでなく、可溶性MDA5としての分泌も促進される可能性が考えられる。これは、全身性のtype I interferonopathyのみならず、抗MDA5抗体の産生を促進する一因となる可能性を示唆している。

1. **Treatment of MDA5-DM**
   1. **Current Treatment Strategies**

現在、MDA5-DMは標準治療として高用量ステロイド、カルシニューリン阻害薬、大量シクロホスファミド静脈投与の3剤併用が併用され、生命予後を改善させる事が示されている(97)。

前述の通り、病態に関わる様々な免疫細胞や、サイトカイン、ケモカインを抑制するためには、あらゆる免疫細胞を抑制する事が可能なグルココルチコイドに加えて、炎症や線維化に関わるCD8^+^T細胞やCD4^+^T細胞を抑制するカルシニューリン阻害薬、さらにはT細胞やB細胞を共に抑制するシクロホスファミドの併用は疾患活動性を抑制する観点で重要である。カルシニューリン阻害薬に関しては、オープンラベルのランダム化比較試験によると、タクロリムスとシクロスポリンAで有意差がなかったという(98)。しかしこの試験でMDA5-DMの患者の割合は3割であった事に留意すべきである。また、タクロリムスに関しては従来の濃度では不足である事が指摘されており、再発予防のためには、他の特発性筋疾患よりも高いタクロリムス濃度が必要で、CYP3A5 遺伝子型に基づくタクロリムス投与量の個別化が推奨されている(99)。

皮膚筋炎に対するIntravenous immune globulin (IVIG)を検討したランダム化比較試験の結果では、有意な臨床的改善を示した(100)。しかしながら、この臨床試験では抗MDA5抗体の陽性率が極めて低かった。一方、MDA5-DMを対象とした後ろ向き観察研究の結果では、他の皮膚筋炎同様に早期のIVIGが有効であったという報告がある(101)。しかしながら、初期治療の3剤併用療法の影響が完全に否定できない事やIVIGの適切な投与間隔が不明である事が課題である。

また、病原性が疑われている血液中の抗MDA5抗体や、肺組織に沈着している補体やIgGなどの免疫グロブリン(85)、炎症性サイトカインを除去できる観点では、ポリミキシン B 固定化繊維カラム血液灌流療法や血漿交換の有用性が報告されている(37,102,103)。一方、51例の患者を含む多施設共同研究では、血漿交換の有効性は示されなかったが(102)、5件の後ろ向きコホート研究、4件のケースコントロール研究、2件のケースシリーズを含むシステマティックレビューの結果では、血漿交換の有用性が期待された(104)。今後、より大規模な研究と適切な実施間隔や期間の決定が必要である。

さらに、MDA5-DMに対する強化免疫抑制療法中に*Pneumocystis jirovecii* pneumoniaが高確率で発生する事が知られている(105,106)。これらに対するtrimethoprim–sulfamethoxazoleによる予防療法がMDA5-DMの予後を改善する事は想像するに容易い(107)。

- 1. **Emerging Therapeutic Options**

　免疫抑制薬に関してはカルシニューリン阻害薬が無効の際に、ミコフェノール酸モフェチルを追加して状態が改善したケースレポートがある(108)。一方で、アザチオプリンやメトトレキサート、レフルノミドは有効性が示されておらず、原則として推奨されない(109)。

　生物学的製剤に関してはほとんど報告がされていない。トシリズマブが有効であった症例報告やケースシリーズはある(110,111)。興味深い事に、MDA5-DMではIL-6が高値の場合に予後不良である事と、MDA5を含むjuvenile dermatomyositisではIL-6/STAT3 signaling pathwayが活性化している事が報告されているが、トシリズマブの使用については現時点では限定的であろう(112,113)。

近年MDA5-DMにおけるJAK阻害薬の有効性が報告されている。JAK阻害薬はMDA5-DMの主病態であるtype I IFNだけでなく、線維化などに関わるtype II IFNやIL-21の抑制にもJAK-STAT経路(JAK1-STAT1/JAK3-STAT5)が関わるため、急性期の炎症を抑制するだけでなく、慢性期の肺線維症にも有効となる可能性がある(114,115)。JAK阻害薬は従来の3剤併用療法に不応の場合に併用される報告が多いが、メタ解析では開始のタイミングに関わらず有効性があるようである(116)。また、JAK阻害薬はクラスに関わらずの有効性を示す(117–120)。

前述の通り、MDA5-DMにおいて抗MDA5抗体の病原性の可能性が示唆されている上、抗Ro抗体陽性が予後因子になる報告や、抗S抗体などの様々な自己抗体が産生される事からは、B細胞の寛容破綻が病態に関わる事が考えられる。そのため、B細胞除去療法の有用性も期待される。一部では報告では、難治性の症例に対してB細胞除去療法であるリツキシマブを通常量(2週毎に1000mgを合計2回あるいは毎週500mgを4回)あるいは低用量(毎週100mgを4回)追加する事が有効であった報告がある(121–124)。さらにリツキシマブが無効の場合でも、形質細胞に高発現するCD38をターゲットとした抗CD38抗体(ダラツマブ)が有効だった症例もある(125–127)。特にMDA5-DMでは、CD38^+^CD4^+^T細胞あるいはCD38^+^CD8^+^T細胞が増加しており、RP-ILDの予後不良と関連しており、今後、それらの特異的なT細胞の抑制治療が期待される(128)。

また、グルココルチコイドとカルシニューリン阻害薬に不応なRP-ILDに対してヒトT細胞のIL-2受容体α鎖(CD25)に対するマウスモノクローナル抗体であるバシリキシマブを使用した4名の患者のケースシリーズでは75%に有効性を示した(129)。しかしながら、最初の発表(2014年)から10年以上経過しているが、その後、追加で有効性を示す報告はない。

T細胞とB細胞の両方をターゲットにする治療薬として、B細胞成熟抗原（BCMA）及びCD3を標的とする二重特異性抗体であるテクリスタマブをMDA5-DMを含む4名の自己免疫疾患患者に投与した報告がある(130)。その結果、4名とも良好な経過を示し、新たな治療選択肢として期待される。

抗線維化薬については早期に使用した場合に有効であったケースシリーズがある(131)。急性期には肺線維症は稀な病態だが、慢性経過の場合は線維化を起こす可能性が高まるため、一部の患者では有効性を示す可能性がある(132)。

肺移植については選択肢として有効性が期待される。11例をまとめたシステマティックレビューによると、10/11人(90.1％)で再発を起こすことなく、経過したという(133)。しかしながら、これらのケースレポートには出版バイアスがある可能性と、適切な施行時期の決定が困難である事を考慮すべきである。

以下に現在の治療選択肢と今後期待される治療選択肢をまとめる(Figure 4)。

**Figure 4. Treatment choice of MDA5-DM**


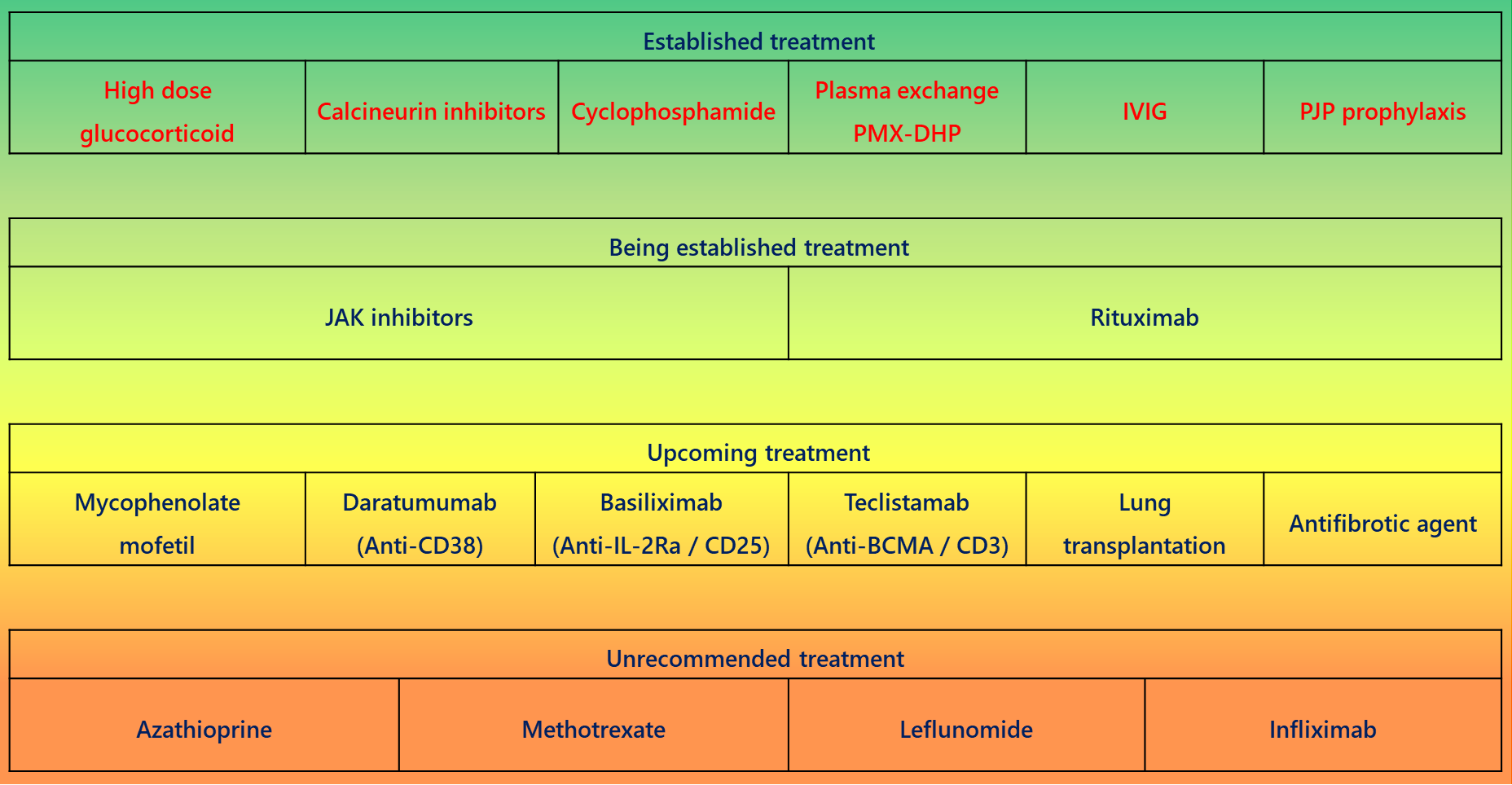


**CONCLUSION**

MDA5-DMは日本人を含めたアジア人に多く、重症度も高い疾患である。近年の研究により、遺伝的病因や環境要因、局所傷害臓器での免疫細胞の挙動、type I interferonopathyとMDA5のポジティブフィードバック機構の可能性、抗MDA5抗体の病原性とサブタイプなど様々な病態が解明されて来た。また、治療に関しては従来の3剤併用療法に加えてJAK阻害薬やリツキシマブなどの新規治療選択肢の報告が蓄積してきた。一方、近年の報告では、MDA5-DMにおいて重症度の異なる複数の臨床フェノタイプが存在することが示唆されている(134–136)。今後、全ての患者で強化免疫抑制療法が必要か再度検討するためにも適切なフェノタイプ分類を行う必要がある。

**REFERENCES**

1. Nakashima R, Hosono Y, Mimori T. Clinical significance and new detection system of autoantibodies in myositis with interstitial lung disease. Lupus. 2016 Jul;25(8):925–33.

2. Lin RR, Warp PV, Maderal AD, Elman SA. Assessing Time of Onset for Interstitial Lung Disease in Anti-MDA5 Antibody-Positive Dermatomyositis. JAMA Dermatol. 2024 May;160(5):575–7.

3. Betteridge Z, Tansley S, Shaddick G, Chinoy H, Cooper RG, New RP, et al. Frequency, mutual exclusivity and clinical associations of myositis autoantibodies in a combined European cohort of idiopathic inflammatory myopathy patients. J Autoimmun. 2019 Jul;101:48–55.

4. Nombel A, Fabien N, Coutant F. Dermatomyositis With Anti-MDA5 Antibodies: Bioclinical Features, Pathogenesis and Emerging Therapies. Front Immunol. 2021;12:773352.

5. Nakashima R, Imura Y, Kobayashi S, Yukawa N, Yoshifuji H, Nojima T, et al. The RIG-I-like receptor IFIH1/MDA5 is a dermatomyositis-specific autoantigen identified by the anti-CADM-140 antibody. Rheumatology (Oxford). 2010 Mar;49(3):433–40.

6. Muro Y, Sugiura K, Hoshino K, Akiyama M, Tamakoshi K. Epidemiologic study of clinically amyopathic dermatomyositis and anti-melanoma differentiation-associated gene 5 antibodies in central Japan. Arthritis Res Ther. 2011;13(6):R214.

7. Koga T, Fujikawa K, Horai Y, Okada A, Kawashiri SY, Iwamoto N, et al. The diagnostic utility of anti-melanoma differentiation-associated gene 5 antibody testing for predicting the prognosis of Japanese patients with DM. Rheumatology (Oxford). 2012 Jul;51(7):1278–84.

8. Sato S, Hirakata M, Kuwana M, Suwa A, Inada S, Mimori T, et al. Autoantibodies to a 140-kd polypeptide, CADM-140, in Japanese patients with clinically amyopathic dermatomyositis. Arthritis Rheum. 2005 May;52(5):1571–6.

9. Ceribelli A, Fredi M, Taraborelli M, Cavazzana I, Tincani A, Selmi C, et al. Prevalence and clinical significance of anti-MDA5 antibodies in European patients with polymyositis/dermatomyositis. Clin Exp Rheumatol. 2014;32(6):891–7.

10. Fujikawa K, Kawakami A, Kaji K, Fujimoto M, Kawashiri S, Iwamoto N, et al. Association of distinct clinical subsets with myositis-specific autoantibodies towards anti-155/140-kDa polypeptides, anti-140-kDa polypeptides, and anti-aminoacyl tRNA synthetases in Japanese patients with dermatomyositis: a single-centre, cross-sectional study. Scand J Rheumatol. 2009;38(4):263–7.

11. Fiorentino D, Chung L, Zwerner J, Rosen A, Casciola-Rosen L. The mucocutaneous and systemic phenotype of dermatomyositis patients with antibodies to MDA5 (CADM-140): a retrospective study. J Am Acad Dermatol. 2011 Jul;65(1):25–34.

12. Labrador-Horrillo M, Martinez MA, Selva-O’Callaghan A, Trallero-Araguas E, Balada E, Vilardell-Tarres M, et al. Anti-MDA5 antibodies in a large Mediterranean population of adults with dermatomyositis. J Immunol Res. 2014;2014:290797.

13. Gono T, Kawaguchi Y, Kuwana M, Sugiura T, Furuya T, Takagi K, et al. Brief report: Association of HLA-DRB1*0101/*0405 with susceptibility to anti-melanoma differentiation-associated gene 5 antibody-positive dermatomyositis in the Japanese population. Arthritis Rheum. 2012 Nov;64(11):3736–40.

14. Rothwell S, Chinoy H, Lamb JA, Miller FW, Rider LG, Wedderburn LR, et al. Focused HLA analysis in Caucasians with myositis identifies significant associations with autoantibody subgroups. Ann Rheum Dis. 2019 Jul;78(7):996–1002.

15. Kochi Y, Kamatani Y, Kondo Y, Suzuki A, Kawakami E, Hiwa R, et al. Splicing variant of WDFY4 augments MDA5 signalling and the risk of clinically amyopathic dermatomyositis. Ann Rheum Dis. 2018 Apr;77(4):602–11.

16. Guo L, Zhang X, Pu W, Zhao J, Wang K, Zhang D, et al. WDFY4 polymorphisms in Chinese patients with anti-MDA5 dermatomyositis is associated with rapid progressive interstitial lung disease. Rheumatology (Oxford). 2023 Jun;62(6):2320–4.

17. Theisen DJ, Davidson JT, Briseño CG, Gargaro M, Lauron EJ, Wang Q, et al. WDFY4 is required for cross-presentation in response to viral and tumor antigens. Science. 2018 Nov;362(6415):694–9.

18. Li Y, Li J, Yuan Q, Bian X, Long F, Duan R, et al. Deficiency in WDFY4 reduces the number of CD8+ T cells via reactive oxygen species-induced apoptosis. Mol Immunol. 2021 Nov;139:131–8.

19. Chen Z, Cao M, Plana MN, Liang J, Cai H, Kuwana M, et al. Utility of anti-melanoma differentiation-associated gene 5 antibody measurement in identifying patients with dermatomyositis and a high risk for developing rapidly progressive interstitial lung disease: a review of the literature and a meta-analysis. Arthritis Care Res (Hoboken). 2013 Aug;65(8):1316–24.

20. Nishina N, Sato S, Masui K, Gono T, Kuwana M. Seasonal and residential clustering at disease onset of anti-MDA5-associated interstitial lung disease. RMD Open. 2020 Jun;6(2):e001202.

21. Toquet S, Granger B, Uzunhan Y, Mariampillai K, Nunes H, Benveniste O, et al. The seasonality of Dermatomyositis associated with anti-MDA5 antibody: An argument for a respiratory viral trigger. Autoimmun Rev. 2021 Apr;20(4):102788.

22. Palterer B, Mazzoni A, Infantino M, Semeraro R, Manfredi M, Pesce G, et al. Seasonal patterns of myositis-specific and myositis-associated autoantibodies in Italy: Seasonal patterns of myositis autoantibodies. Immunol Lett. 2024 Dec;272:106966.

23. So H, So J, Lam TTO, Wong VTL, Ho R, Li WL, et al. Seasonal Effect on Disease Onset and Presentation in Anti-MDA5 Positive Dermatomyositis. Front Med (Lausanne). 2022;9:837024.

24. Kang D chul, Gopalkrishnan RV, Wu Q, Jankowsky E, Pyle AM, Fisher PB. mda-5: An interferon-inducible putative RNA helicase with double-stranded RNA-dependent ATPase activity and melanoma growth-suppressive properties. Proc Natl Acad Sci U S A. 2002 Jan;99(2):637–42.

25. Kang DC, Gopalkrishnan RV, Lin L, Randolph A, Valerie K, Pestka S, et al. Expression analysis and genomic characterization of human melanoma differentiation associated gene-5, mda-5: a novel type I interferon-responsive apoptosis-inducing gene. Oncogene. 2004 Mar;23(9):1789–800.

26. Zahn S, Barchet W, Rehkämper C, Hornung T, Bieber T, Tüting T, et al. Enhanced skin expression of melanoma differentiation-associated gene 5 (MDA5) in dermatomyositis and related autoimmune diseases. J Am Acad Dermatol. 2011 May;64(5):988–9.

27. Gono T, Okazaki Y, Kuwana M. Antiviral proinflammatory phenotype of monocytes in anti-MDA5 antibody-associated interstitial lung disease. Rheumatology (Oxford). 2022 Feb;61(2):806–14.

28. Liu Y, Feng S, Liu X, Tang Y, Li X, Luo C, et al. IFN-beta and EIF2AK2 are potential biomarkers for interstitial lung disease in anti-MDA5 positive dermatomyositis. Rheumatology (Oxford). 2023 Nov;62(11):3724–31.

29. Jayaraman S, Tiniakou E, Morgenlander WR, Na M, Christopher-Stine L, Larman HB. Comprehensive Enteroviral Serology Links Infection and Anti-Melanoma Differentiation-Associated Protein 5 Dermatomyositis. ACR Open Rheumatol. 2024 Nov;

30. Castellini C, Scotti C, Navarini L, Fu Q, Qian J, Giacomelli R, et al. The evaluation of type I interferon score in dermatomyositis, a systematic review and a meta-analysis. Autoimmun Rev. 2024 Dec;23(12):103686.

31. Zhang SH, Zhao Y, Xie QB, Jiang Y, Wu YK, Yan B. Aberrant activation of the type I interferon system may contribute to the pathogenesis of anti-melanoma differentiation-associated gene 5 dermatomyositis. Br J Dermatol. 2019 May;180(5):1090–8.

32. Cassius C, Amode R, Delord M, Battistella M, Poirot J, How-Kit A, et al. MDA5+ Dermatomyositis Is Associated with Stronger Skin Type I Interferon Transcriptomic Signature with Upregulation of IFN-κ Transcript. J Invest Dermatol. 2020 Jun;140(6):1276-1279.e7.

33. Ye Y, Chen Z, Jiang S, Jia F, Li T, Lu X, et al. Single-cell profiling reveals distinct adaptive immune hallmarks in MDA5+ dermatomyositis with therapeutic implications. Nat Commun. 2022 Oct;13(1):6458.

34. Ono N, Kai K, Maruyama A, Sakai M, Sadanaga Y, Koarada S, et al. The relationship between type 1 IFN and vasculopathy in anti-MDA5 antibody-positive dermatomyositis patients. Rheumatology (Oxford). 2019 May;58(5):786–91.

35. Qian J, Li R, Chen Z, Cao Z, Lu L, Fu Q. Type I interferon score is associated with the severity and poor prognosis in anti-MDA5 antibody-positive dermatomyositis patients. Front Immunol. 2023;14:1151695.

36. Funabiki M, Kato H, Miyachi Y, Toki H, Motegi H, Inoue M, et al. Autoimmune disorders associated with gain of function of the intracellular sensor MDA5. Immunity. 2014 Feb;40(2):199–212.

37. Shirakashi M, Nakashima R, Tsuji H, Tanizawa K, Handa T, Hosono Y, et al. Efficacy of plasma exchange in anti-MDA5-positive dermatomyositis with interstitial lung disease under combined immunosuppressive treatment. Rheumatology (Oxford). 2020 Nov 1;59(11):3284–92.

38. Fujisawa T, Hozumi H, Yasui H, Suzuki Y, Karayama M, Furuhashi K, et al. Clinical Significance of Serum Chitotriosidase Level in Anti-MDA5 Antibody-positive Dermatomyositis-associated Interstitial Lung Disease. J Rheumatol. 2019 Aug;46(8):935–42.

39. Enomoto Y, Suzuki Y, Hozumi H, Mori K, Kono M, Karayama M, et al. Clinical significance of soluble CD163 in polymyositis-related or dermatomyositis-related interstitial lung disease. Arthritis Res Ther. 2017 Jan;19(1):9.

40. Liang L, Zhang YM, Shen YW, Song AP, Li WL, Ye LF, et al. Aberrantly Expressed Galectin-9 Is Involved in the Immunopathogenesis of Anti-MDA5-Positive Dermatomyositis-Associated Interstitial Lung Disease. Front Cell Dev Biol. 2021;9:628128.

41. Kokuzawa A, Nakamura J, Kamata Y, Sato K. Potential role of type I interferon/IP-10 axis in the pathogenesis of anti-MDA5 antibody-positive dermatomyositis. Clin Exp Rheumatol. 2023 Mar;41(2):275–84.

42. Kuzumi A, Fukasawa T, Yamashita T, Matsuda KM, Kotani H, Yoshizaki-Ogawa A, et al. Serum interleukin-34 levels in dermatomyositis: a potential biomarker for anti-MDA5-antibody-associated interstitial lung disease. Rheumatology (Oxford). 2024 Jun;keae313.

43. Gono T, Miyake K, Kawaguchi Y, Kaneko H, Shinozaki M, Yamanaka H. Hyperferritinaemia and macrophage activation in a patient with interstitial lung disease with clinically amyopathic DM. Rheumatology (Oxford). 2012 Jul;51(7):1336–8.

44. Shi J, Pei X, Peng J, Wu C, Lv Y, Wang X, et al. Monocyte-macrophage dynamics as key in disparate lung and peripheral immune responses in severe anti-melanoma differentiation-associated gene 5-positive dermatomyositis-related interstitial lung disease. Clin Transl Med. 2025 Feb;15(2):e70226.

45. Gono T, Kaneko H, Kawaguchi Y, Hanaoka M, Kataoka S, Kuwana M, et al. Cytokine profiles in polymyositis and dermatomyositis complicated by rapidly progressive or chronic interstitial lung disease. Rheumatology (Oxford). 2014 Dec;53(12):2196–203.

46. Thuner J, Coutant F. IFN-γ: An overlooked cytokine in dermatomyositis with anti-MDA5 antibodies. Autoimmun Rev. 2023 Oct;22(10):103420.

47. Fukada A, Fujisawa T, Hozumi H, Koda K, Akamatsu T, Oyama Y, et al. Prognostic Role of Interferon-λ3 in Anti-Melanoma Differentiation-Associated Gene 5-Positive Dermatomyositis-Associated Interstitial Lung Disease. Arthritis Rheumatol. 2024 May;76(5):796–805.

48. Ishikawa Y, Iwata S, Hanami K, Nawata A, Zhang M, Yamagata K, et al. Relevance of interferon-gamma in pathogenesis of life-threatening rapidly progressive interstitial lung disease in patients with dermatomyositis. Arthritis Res Ther. 2018 Oct;20(1):240.

49. Coutant F, Bachet R, Pin JJ, Alonzo M, Miossec P. Monoclonal antibodies from B cells of patients with anti-MDA5 antibody-positive dermatomyositis directly stimulate interferon gamma production. J Autoimmun. 2022 Jun;130:102831.

50. Ishida Y, Kimura A, Nosaka M, Kuninaka Y, Hemmi H, Sasaki I, et al. Essential involvement of the CX3CL1-CX3CR1 axis in bleomycin-induced pulmonary fibrosis via regulation of fibrocyte and M2 macrophage migration. Sci Rep. 2017 Dec;7(1):16833.

51. Isozaki T, Otsuka K, Sato M, Takahashi R, Wakabayashi K, Yajima N, et al. Synergistic induction of CX3CL1 by interleukin-1β and interferon-γ in human lung fibroblasts: involvement of signal transducer and activator of transcription 1 signaling pathways. Transl Res. 2011 Feb;157(2):64–70.

52. Tsoi LC, Gharaee-Kermani M, Berthier CC, Nault T, Hile GA, Estadt SN, et al. IL18-containing 5-gene signature distinguishes histologically identical dermatomyositis and lupus erythematosus skin lesions. JCI Insight. 2020 Aug;5(16):e139558, 139558.

53. Wen J, Zhou M, Lai Y, Zhuang L, Shi J, Lin Z, et al. Serum level of IFN-λ is elevated in idiopathic inflammatory myopathies. Clin Rheumatol. 2024 Nov;

54. Huang W, Ren F, Luo L, Zhou J, Huang D, Pan Z, et al. The characteristics of lymphocytes in patients positive for anti-MDA5 antibodies in interstitial lung disease. Rheumatology (Oxford). 2020 Dec;59(12):3886–91.

55. Chen F, Wang D, Shu X, Nakashima R, Wang G. Anti-MDA5 antibody is associated with A/SIP and decreased T cells in peripheral blood and predicts poor prognosis of ILD in Chinese patients with dermatomyositis. Rheumatol Int. 2012 Dec;32(12):3909–15.

56. Jin Q, Fu L, Yang H, Chen X, Lin S, Huang Z, et al. Peripheral lymphocyte count defines the clinical phenotypes and prognosis in patients with anti-MDA5-positive dermatomyositis. J Intern Med. 2023 Apr;293(4):494–507.

57. Tian Y, He P, Ren L, Xin H, Xi B, Zou R, et al. Dynamic change of lymphocytes associated with short-term prognosis in anti-MDA5-positive dermatomyositis with interstitial lung disease: a multicenter retrospective study. Clin Rheumatol. 2024 Sep;

58. Zhang L, Xia Q, Li W, Peng Q, Yang H, Lu X, et al. The RIG-I pathway is involved in peripheral T cell lymphopenia in patients with dermatomyositis. Arthritis Res Ther. 2019 May;21(1):131.

59. Matsushita T, Kobayashi T, Kano M, Hamaguchi Y, Takehara K. Elevated serum B-cell activating factor levels in patients with dermatomyositis: Association with interstitial lung disease. J Dermatol. 2019 Dec;46(12):1190–6.

60. Shi Y, You H, Liu C, Qiu Y, Lv C, Zhu Y, et al. Elevated serum B-cell activator factor levels predict rapid progressive interstitial lung disease in anti-melanoma differentiation associated protein 5 antibody positive dermatomyositis. Orphanet J Rare Dis. 2024 Apr;19(1):170.

61. Wang Y, Zhu L, Ju B, Luo J, Li Q, Lv X, et al. Alterations of peripheral blood B cell subsets in Chinese patients with adult idiopathic inflammatory myopathies. Clin Exp Rheumatol. 2022 Feb;40(2):260–6.

62. Sugimori Y, Iwasaki Y, Takeshima Y, Okubo M, Kobayashi S, Hatano H, et al. Transcriptome Profiling of Immune Cell Types in Peripheral Blood Reveals Common and Specific Pathways Involved in the Pathogenesis of Myositis-Specific Antibody-Positive Inflammatory Myopathies. ACR Open Rheumatol. 2023 Feb;5(2):93–102.

63. Lv C, You H, Xu L, Wang L, Yuan F, Li J, et al. Coexistence of Anti-Ro52 Antibodies in Anti-MDA5 Antibody-Positive Dermatomyositis Is Highly Associated With Rapidly Progressive Interstitial Lung Disease and Mortality Risk. J Rheumatol. 2023 Feb;50(2):219–26.

64. Xu A, Ye Y, Fu Q, Lian X, Chen S, Guo Q, et al. Prognostic values of anti-Ro52 antibodies in anti-MDA5-positive clinically amyopathic dermatomyositis associated with interstitial lung disease. Rheumatology (Oxford). 2021 Jul;60(7):3343–51.

65. Gui X, Shenyun S, Ding H, Wang R, Tong J, Yu M, et al. Anti-Ro52 antibodies are associated with the prognosis of adult idiopathic inflammatory myopathy-associated interstitial lung disease. Rheumatology (Oxford). 2022 Nov;61(11):4570–8.

66. Hosono Y, Nakashima R, Serada S, Murakami K, Imura Y, Yoshifuji H, et al. Splicing factor proline/glutamine-rich is a novel autoantigen of dermatomyositis and associated with anti-melanoma differentiation-associated gene 5 antibody. J Autoimmun. 2017 Feb;77:116–22.

67. Decker P, Moulinet T, Pontille F, Cravat M, De Carvalho Bittencourt M, Jaussaud R. An updated review of anti-Ro52 (TRIM21) antibodies impact in connective tissue diseases clinical management. Autoimmun Rev. 2022 Mar;21(3):103013.

68. Higgs R, Ní Gabhann J, Ben Larbi N, Breen EP, Fitzgerald KA, Jefferies CA. The E3 ubiquitin ligase Ro52 negatively regulates IFN-beta production post-pathogen recognition by polyubiquitin-mediated degradation of IRF3. J Immunol. 2008 Aug;181(3):1780–6.

69. Higgs R, Lazzari E, Wynne C, Ní Gabhann J, Espinosa A, Wahren-Herlenius M, et al. Self protection from anti-viral responses--Ro52 promotes degradation of the transcription factor IRF7 downstream of the viral Toll-Like receptors. PLoS One. 2010 Jul;5(7):e11776.

70. Espinosa A, Hennig J, Ambrosi A, Anandapadmanaban M, Abelius MS, Sheng Y, et al. Anti-Ro52 autoantibodies from patients with Sjögren’s syndrome inhibit the Ro52 E3 ligase activity by blocking the E3/E2 interface. J Biol Chem. 2011 Oct;286(42):36478–91.

71. Jiang Y, Liu Y, Zhao Y, Zheng Y, Yu M, Deng J, et al. Mitochondrial morphology and MAVS-IFN1 signaling pathway in muscles of anti-MDA5 dermatomyositis. Ann Clin Transl Neurol. 2021 Mar;8(3):677–86.

72. Li C, Han Y, Li X, Zhang H, Yao Z, Zhou J, et al. Soluble CXCL16 is a prognostic biomarker associated with rapidly progressive interstitial lung disease complicated with dermatomyositis. Semin Arthritis Rheum. 2024 Aug;67:152483.

73. Zuo Y, Ye L, Liu M, Li S, Liu W, Chen F, et al. Clinical significance of radiological patterns of HRCT and their association with macrophage activation in dermatomyositis. Rheumatology (Oxford). 2020 Oct;59(10):2829–37.

74. Wang K, Zhao J, Chen Z, Li T, Tan X, Zheng Y, et al. CD4+CXCR4+ T cells as a novel prognostic biomarker in patients with idiopathic inflammatory myopathy-associated interstitial lung disease. Rheumatology (Oxford). 2019 Mar;58(3):511–21.

75. Brodeur TY, Robidoux TE, Weinstein JS, Craft J, Swain SL, Marshak-Rothstein A. IL-21 Promotes Pulmonary Fibrosis through the Induction of Profibrotic CD8+ T Cells. J Immunol. 2015 Dec;195(11):5251–60.

76. Zhu L, Xu Y, Wang J, Zhang Y, Zhou J, Wu H. Mesenchymal stem cells-derived exosomes carrying microRNA-30b confer protection against pulmonary fibrosis by downregulating Runx1 via Spred2. Mol Genet Genomics. 2024 Mar;299(1):33.

77. Shen N, Zhou X, Jin X, Lu C, Hu X, Zhang Y, et al. MDA5 expression is associated with TGF-β-induced fibrosis: potential mechanism of interstitial lung disease in anti-MDA5 dermatomyositis. Rheumatology (Oxford). 2022 Dec;62(1):373–83.

78. Wang Y, Jia H, Li W, Liu H, Tu M, Li J, et al. Transcriptomic profiling and longitudinal study reveal the relationship of anti-MDA5 titer and type I IFN signature in MDA5+ dermatomyositis. Front Immunol. 2023;14:1249844.

79. Matsushita T, Mizumaki K, Kano M, Yagi N, Tennichi M, Takeuchi A, et al. Antimelanoma differentiation-associated protein 5 antibody level is a novel tool for monitoring disease activity in rapidly progressive interstitial lung disease with dermatomyositis. Br J Dermatol. 2017 Feb;176(2):395–402.

80. Muro Y, Sugiura K, Hoshino K, Akiyama M. Disappearance of anti-MDA-5 autoantibodies in clinically amyopathic DM/interstitial lung disease during disease remission. Rheumatology (Oxford). 2012 May;51(5):800–4.

81. Abe Y, Matsushita M, Tada K, Yamaji K, Takasaki Y, Tamura N. Clinical characteristics and change in the antibody titres of patients with anti-MDA5 antibody-positive inflammatory myositis. Rheumatology (Oxford). 2017 Sep;56(9):1492–7.

82. Seto N, Torres-Ruiz JJ, Carmona-Rivera C, Pinal-Fernandez I, Pak K, Purmalek MM, et al. Neutrophil dysregulation is pathogenic in idiopathic inflammatory myopathies. JCI Insight. 2020 Feb;5(3):e134189, 134189.

83. Wang K, Zhao J, Wu W, Xu W, Sun S, Chen Z, et al. RNA-Containing Immune Complexes Formed by Anti-Melanoma Differentiation Associated Gene 5 Autoantibody Are Potent Inducers of IFN-α. Front Immunol. 2021;12:743704.

84. Pinal-Fernandez I, Muñoz-Braceras S, Casal-Dominguez M, Pak K, Torres-Ruiz J, Musai J, et al. Pathological autoantibody internalisation in myositis. Ann Rheum Dis. 2024 Jun;ard-2024-225773.

85. Zaizen Y, Okamoto M, Azuma K, Fukuoka J, Hozumi H, Sakamoto N, et al. Enhanced immune complex formation in the lungs of patients with dermatomyositis. Respir Res. 2023 Mar;24(1):86.

86. Ichimura Y, Konishi R, Shobo M, Tanaka R, Kubota N, Kayama H, et al. Autoimmunity against melanoma differentiation-associated gene 5 induces interstitial lung disease mimicking dermatomyositis in mice. Proc Natl Acad Sci U S A. 2024 Apr;121(16):e2313070121.

87. Chen M, Zhao Q, Diao L, Xue K, Ruan Y, Xue F, et al. Distribution of anti-melanoma differentiation associated gene 5 (MDA5) IgG subclasses in MDA5+ dermatomyositis. Rheumatology (Oxford). 2021 Dec;61(1):430–9.

88. Xu YT, Zhang YM, Yang HX, Ye LF, Chen F, Lu X, et al. Evaluation and validation of the prognostic value of anti-MDA5 IgG subclasses in dermatomyositis-associated interstitial lung disease. Rheumatology (Oxford). 2022 Dec;62(1):397–406.

89. Hoepel W, Allahverdiyeva S, Harbiye H, de Taeye SW, van der Ham AJ, de Boer L, et al. IgG Subclasses Shape Cytokine Responses by Human Myeloid Immune Cells through Differential Metabolic Reprogramming. J Immunol. 2020 Dec;205(12):3400–7.

90. Mo Y, Ye Y, Peng L, Sun X, Zhong X, Wu R. The central helicase domain holds the major conformational epitopes of melanoma differentiation-associated gene 5 autoantibodies. Rheumatology (Oxford). 2024 May;63(5):1456–65.

91. Van Gompel E, Demirdal D, Fernandes-Cerqueira C, Horuluoglu B, Galindo-Feria A, Wigren E, et al. Autoantibodies against the melanoma differentiation-associated protein 5 in patients with dermatomyositis target the helicase domains. Rheumatology (Oxford). 2024 May;63(5):1466–73.

92. Yamaguchi K, Poland P, Zhu L, Moghadam-Kia S, Aggarwal R, Maeno T, et al. Comparative B cell epitope profiling in Japanese and North American cohorts of MDA5+ dermatomyositis reveals a direct association between immune repertoire and pulmonary mortality. Rheumatology (Oxford). 2024 Aug;keae466.

93. Yamaguchi K, Poland P, Bijoy George T, Saygin D, Moghadam-Kia S, Aggarwal R, et al. Correlation between B-cell epitope profile and clinical features of anti-MDA5 antibody-positive dermatomyositis. Rheumatology (Oxford). 2024 Jul;63(7):2016–23.

94. Hara R, Watanabe S, Terada N, Kase K, Muto A, Hamaguchi Y, et al. The lung as a site for the generation of anti-MDA5 antibody in clinically amyopathic dermatomyositis. Rheumatology (Oxford). 2024 Jun;keae314.

95. Tamaki M, Matsumi S, Nakasone H, Nakamura Y, Kawamura M, Kawamura S, et al. Interstitial lung disease with anti-melanoma differentiation-associated gene 5 antibody after allogeneic hematopoietic stem cell transplantation. Bone Marrow Transplant. 2022 Sep;57(9):1382–8.

96. Okamoto M, Zaizen Y, Kaieda S, Nouno T, Koga T, Matama G, et al. Soluble form of the MDA5 protein in human sera. Heliyon. 2024 Jun;10(11):e31727.

97. Tsuji H, Nakashima R, Hosono Y, Imura Y, Yagita M, Yoshifuji H, et al. Multicenter Prospective Study of the Efficacy and Safety of Combined Immunosuppressive Therapy With High-Dose Glucocorticoid, Tacrolimus, and Cyclophosphamide in Interstitial Lung Diseases Accompanied by Anti-Melanoma Differentiation-Associated Gene 5-Positive Dermatomyositis. Arthritis Rheumatol. 2020 Mar;72(3):488–98.

98. Fujisawa T, Hozumi H, Kamiya Y, Kaida Y, Akamatsu T, Kusagaya H, et al. Prednisolone and tacrolimus versus prednisolone and cyclosporin A to treat polymyositis/dermatomyositis-associated ILD: A randomized, open-label trial. Respirology. 2021 Apr;26(4):370–7.

99. Tian X, Liu L, Liu S, Yang J. Tacrolimus Personalized Therapy based on CYP3A5 Genotype in Chinese Patients with Idiopathic Inflammatory Myopathies. Rheumatology (Oxford). 2024 Jun;keae316.

100. Aggarwal R, Charles-Schoeman C, Schessl J, Bata-Csörgő Z, Dimachkie MM, Griger Z, et al. Trial of Intravenous Immune Globulin in Dermatomyositis. N Engl J Med. 2022 Oct;387(14):1264–78.

101. Wang LM, Yang QH, Zhang L, Liu SY, Zhang PP, Zhang X, et al. Intravenous immunoglobulin for interstitial lung diseases of anti-melanoma differentiation-associated gene 5-positive dermatomyositis. Rheumatology (Oxford). 2022 Aug;61(9):3704–10.

102. Bay P, de Chambrun MP, Rothstein V, Mahevas M, De Prost N, Roux A, et al. Efficacy of plasma exchange in patients with anti-MDA5 rapidly progressive interstitial lung disease. J Autoimmun. 2022 Dec;133:102941.

103. Saito T, Mizobuchi M, Miwa Y, Sugiyama M, Mima Y, Iida A, et al. Anti-MDA-5 antibody-positive clinically amyopathic dermatomyositis with rapidly progressive interstitial lung disease treated with therapeutic plasma exchange: A case series. J Clin Apher. 2021 Feb;36(1):196–205.

104. Yang Y, Yang YT, Huo RX, Meng DL, Huang XX, Lin JY. Short-term efficiency of plasma exchange in combination with immunosuppressants and/or biologics in the treatment of idiopathic inflammatory myopathy with rapidly progressive interstitial lung disease: a systematic review and meta-analysis. Ann Med. 2024 Dec;56(1):2411605.

105. Li J, Wang S, Zheng J, Li Q, Li J, Lu L. Clinical characteristics of and risk factors for Pneumocystis jirovecii pneumonia in anti-melanoma differentiation-associated gene 5 (Anti-MDA5) antibody-positive dermatomyositis patients: a single-center retrospective study. Clin Rheumatol. 2023 Feb;42(2):453–62.

106. Chen X, Shu X, He L, Yang H, Lu X, Wang G, et al. High prevalence and mortality of Pneumocystis jirovecii pneumonia in anti-MDA5 antibody-positive dermatomyositis. Rheumatology (Oxford). 2023 Oct;62(10):3302–9.

107. Liu L, Zhang Y, Liu S, Wang C, Zhang L, Guan W, et al. Compounded sulfamethoxazole improved the prognosis of dermatomyositis patients positive with anti-melanoma differentiation-associated gene 5. Rheumatology (Oxford). 2023 Sep;62(9):3095–100.

108. Hayashi M, Aoki A, Asakawa K, Sakagami T, Kikuchi T, Takada T. Cytokine profiles of amyopathic dermatomyositis with interstitial lung diseases treated with mycophenolate. Respirol Case Rep. 2017 Jul;5(4):e00235.

109. Romero-Bueno F, Diaz Del Campo P, Trallero-Araguás E, Ruiz-Rodríguez JC, Castellvi I, Rodriguez-Nieto MJ, et al. Recommendations for the treatment of anti-melanoma differentiation-associated gene 5-positive dermatomyositis-associated rapidly progressive interstitial lung disease. Semin Arthritis Rheum. 2020 Aug;50(4):776–90.

110. Zhang X, Zhou S, Wu C, Li M, Wang Q, Zhao Y, et al. Tocilizumab for refractory rapidly progressive interstitial lung disease related to anti-MDA5-positive dermatomyositis. Rheumatology (Oxford). 2021 Jul;60(7):e227–8.

111. Qiu L, Shao X, Ma L, Fan Z, Yu H. Successful tocilizumab treatment for rapidly progressive interstitial lung disease with anti-MDA5-positive juvenile dermatomyositis: a case report and literature review. Front Pediatr. 2024;12:1497168.

112. Zheng Q, Wang Z, Tan Y, Zhu K, Lu M. Over Activation of IL-6/STAT3 Signaling Pathway in Juvenile Dermatomyositis. Rheumatol Ther. 2024 Jul;

113. Niu Y, Liu S, Qiu Q, Fu D, Xiao Y, Liang L, et al. Increased serum level of IL-6 predicts poor prognosis in anti-MDA5-positive dermatomyositis with rapidly progressive interstitial lung disease. Arthritis Res Ther. 2024 Oct;26(1):184.

114. Habib T, Senadheera S, Weinberg K, Kaushansky K. The common gamma chain (gamma c) is a required signaling component of the IL-21 receptor and supports IL-21-induced cell proliferation via JAK3. Biochemistry. 2002 Jul;41(27):8725–31.

115. Asao H, Okuyama C, Kumaki S, Ishii N, Tsuchiya S, Foster D, et al. Cutting edge: the common gamma-chain is an indispensable subunit of the IL-21 receptor complex. J Immunol. 2001 Jul;167(1):1–5.

116. Wang Y, Zou R, Wei J, Tang C, Wang J, Lin M. The efficacy and safety of tofacitinib in anti-melanoma differentiation-associated gene 5 antibody positive dermatomyositis associated interstitial lung disease: a systematic review and meta-analysis. Ther Adv Respir Dis. 2024;18:17534666241294000.

117. Chen Z, Wang X, Ye S. Tofacitinib in Amyopathic Dermatomyositis-Associated Interstitial Lung Disease. N Engl J Med. 2019 Jul;381(3):291–3.

118. Ida T, Furuta S, Takayama A, Tamura J, Hayashi Y, Abe K, et al. Efficacy and safety of dose escalation of tofacitinib in refractory anti-MDA5 antibody-positive dermatomyositis. RMD Open. 2023 Jan;9(1):e002795.

119. Huang X, Zhang G, Luo S. A case of refractory anti-MDA5-positive amyopathic dermatomyositis successfully treated with upadacitinib. J Dermatolog Treat. 2024 Dec;35(1):2391445.

120. Harada H, Shoda H, Tsuchiya H, Misaki M, Sawada T, Fujio K. Baricitinib for anti-melanoma differentiation-associated protein 5 antibody-positive dermatomyositis-associated interstitial lung disease: a case series and literature review on Janus kinase inhibitors for the disease. Rheumatol Int. 2024 May;44(5):961–71.

121. Ge Y, Li S, Tian X, He L, Lu X, Wang G. Anti-melanoma differentiation-associated gene 5 (MDA5) antibody-positive dermatomyositis responds to rituximab therapy. Clin Rheumatol. 2021 Jun;40(6):2311–7.

122. Clottu A, Laffitte E, Prins C, Chizzolini C. Response of mucocutaneous lesions to rituximab in a case of melanoma differentiation antigen 5-related dermatomyositis. Dermatology. 2012;225(4):376–80.

123. Koichi Y, Aya Y, Megumi U, Shunichi K, Masafumi S, Hiroaki M, et al. A case of anti-MDA5-positive rapidly progressive interstitial lung disease in a patient with clinically amyopathic dermatomyositis ameliorated by rituximab, in addition to standard immunosuppressive treatment. Mod Rheumatol. 2017 May;27(3):536–40.

124. Mao MM, Xia S, Guo BP, Qian WP, Zheng ZX, Peng XM, et al. Ultra-low dose rituximab as add-on therapy in anti-MDA5-positive patients with polymyositis /dermatomyositis associated ILD. Respir Med. 2020 Oct;172:105983.

125. Holzer MT, Nies JF, Oqueka T, Huber TB, Kötter I, Krusche M. Successful Rescue Therapy With Daratumumab in Rapidly Progressive Interstitial Lung Disease Caused by MDA5-Positive Dermatomyositis. Chest. 2023 Jan;163(1):e1–5.

126. Ostendorf L, Muench F, Thormählen L, Galbavý Z, Körner R, Nee J, et al. Rescue combination treatment of anti-MDA5-associated ARDS with daratumumab. RMD Open. 2023 Jul;9(3):e003238.

127. Chua CG, Chai GT, Lim XR, Manghani M, Leung BPL, Koh LW. Successful rescue treatment of refractory anti-MDA5 autoantibody positive dermatomyositis with rapidly progressive interstitial lung disease using daratumumab. Clin Exp Rheumatol. 2024 Feb;42(2):460–1.

128. Guo Y, Liu H, Chen B, Zhang K, Meng L, Yan L, et al. Dysregulated CD38 expression on T cells was associated with rapidly progressive interstitial lung disease in anti-melanoma differentiation-associated gene 5 positive dermatomyositis. Front Immunol. 2024;15:1455944.

129. Zou J, Li T, Huang X, Chen S, Guo Q, Bao C. Basiliximab may improve the survival rate of rapidly progressive interstitial pneumonia in patients with clinically amyopathic dermatomyositis with anti-MDA5 antibody. Ann Rheum Dis. 2014 Aug;73(8):1591–3.

130. Hagen M, Bucci L, Böltz S, Nöthling DM, Rothe T, Anoshkin K, et al. BCMA-Targeted T-Cell-Engager Therapy for Autoimmune Disease. N Engl J Med. 2024 Sep;391(9):867–9.

131. Bando T, Yamano Y, Takei R, Sasano H, Fukihara J, Yokoyama T, et al. Early intervention of antifibrotics along with anti-inflammatory treatment in rapidly progressive interstitial lung disease (RP-ILD) with anti-MDA5 antibody-positive dermatomyositis (MDA5-DM). European Respiratory Journal [Internet]. 2023 Sep 9 [cited 2024 Jan 30];62(suppl 67). Available from: https://erj.ersjournals.com/content/62/suppl_67/PA401

132. Puthumana RM, Koch AL, Schettino C, Vehar SJ. Asymptomatic and slowly progressive anti-MDA5 ILD: A report of three cases deviating from a notoriously rapidly progressive ILD. Respir Med Case Rep. 2024;51:102072.

133. Lian QY, Chen A, Zhang JH, Xu X, Huang DX, Luo Q, et al. Lung transplantation for anti-MDA5-positive dermatomyositis-associated rapid progressive interstitial lung disease: report of two cases and review of the literature. Clin Rheumatol. 2023 Mar;42(3):941–7.

134. Allenbach Y, Uzunhan Y, Toquet S, Leroux G, Gallay L, Marquet A, et al. Different phenotypes in dermatomyositis associated with anti-MDA5 antibody: Study of 121 cases. Neurology. 2020 Jul;95(1):e70–8.

135. Xu L, You H, Wang L, Lv C, Yuan F, Li J, et al. Identification of Three Different Phenotypes in Anti-Melanoma Differentiation-Associated Gene 5 Antibody-Positive Dermatomyositis Patients: Implications for Prediction of Rapidly Progressive Interstitial Lung Disease. Arthritis Rheumatol. 2023 Apr;75(4):609–19.

136. Guo R, Yang Y, Gu L, Li X, Ma Y, Liu X, et al. Disease-associated immune cell endotypes in anti-MDA5-positive dermatomyositis using unbiased hierarchical clustering. Front Immunol. 2024;15:1349611.
